# Metal Coordination Properties of a Chromophoric Desferrioxamine (DFO) Derivative: Insight on the Coordination Stoichiometry and Thermodynamic Stability of Zr^4+^ Complexes

**DOI:** 10.3390/molecules27010184

**Published:** 2021-12-29

**Authors:** Matteo Savastano, Francesca Boscaro, Antonio Bianchi

**Affiliations:** 1 Department of Chemistry “Ugo Schiff”, Via della Lastruccia 3, 50019 Sesto Fiorentino, Italy; antonio.bianchi@unifi.it; 2 Centro Interdipartimentale di Spettrometria di Massa (CISM), University of Florence, Viale G. Pieraccini 6, 50139 Florence, Italy; francesca.boscaro@unifi.it

**Keywords:** zirconium, desferrioxamine, metal complexes, stability constants, potentiometry, spectrophotometry, mass spectrometry

## Abstract

Desferrioxamine (DFO) is the current “gold standard” chelator for ^89^Zr^4+^, which is used to label monoclonal antibodies for applications in immunopositron emission tomography. Recently, controversial data have been reported regarding the speciation and the stability of the complexes formed by DFO with Zr^4+^ in solution. To shed some light on this point, we studied the coordination properties in solution ofa chromophoric DFO derivative bearing a substituted pyrimidine residue (DFO*–*Pm) toward several metal ions (Zr^4+^, Cu^2+^, Zn^2+^, Mg^2+^, Ca^2+^, Na^+^, K^+^). Potentiometric titrations showed that DFO*–*Pm and pristine DFO form complexes with very similar stoichiometry and stability. DFO*–*Pm, which can consequently be taken as a model system for DFO, provides a photochemical response to metal coordination that can be used to further define the complexes formed. In the critical case of Zr^4+^, spectrophotometric measurements allowed the verification of the formation of 1:1 and 2:3 complexes that, together with 2:2 complexes form the coordination model that was obtained through the use of our potentiometric measurements. Additionally, mass spectrometry measurements verified the formation of 1:1 and 2:3 complexes and showed that 1:2 species can be easily generated through the fragmentation of the 2:3 species. In conclusion, the results obtained with DFO*–*Pm validate the complexation model of Zr^4+^/DFO composed of 1:1, 2:2, and 2:3 metal-to-ligand complexes. Convergences and conflicts with other works are addressed.

## 1. Introduction

Zirconium-89 is one of the most studied radioactive isotopes (in its cationic ^89^Zr^4+^ form) for medical diagnostics using positron emission tomography (PET) and for cancer therapy. The application of this radioactive cation for in vivo imaging or therapy requires it is coordinated to a biologically active targeting molecule with marked site-specificity and high resistance to demetallation/transmetallation processes. Site-specificity is governed by the target vector (e.g., peptides, nucleotides, antibodies, etc.), which is covalently linked to a chelating unit that ensures metal ion sequestration, even at very low concentrations of the radioisotope (10–100 nM), thanks to the high thermodynamic and kinetic stability of the complex [1,2,3].

**Figure 1:**
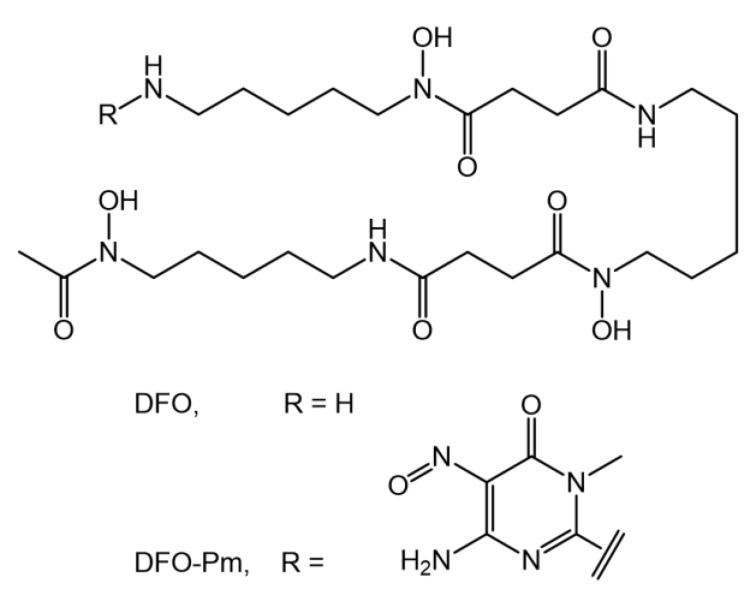
Desferrioxamine (DFO) and its pyrimidine derivative (DFO*–*Pm).

Desferrioxamine (DFO, Figure 1) is the current “gold standard” chelator for ^89^Zr^4+^ [2]. It contains three hydroxamate groups that enforce the formation of very stable complexes with hard metal ions, such as Zr^4+^, and possesses an amine tail that can be easily conjugated to the target vectors. Accordingly, DFO derivatives have so far been the only ^89^Zr^4+^-based agents used in clinical trials [4]. Nevertheless, DFO does not appear to be the optimal chelator for ^89^Zr^4+^ in similar applications, as the in vivo transfer of this radioactive cation to bones has been documented [3,5].

It is accepted that the Zr^4+^ complex of DFO in water contains the metal ion in an octa-coordinated environment of oxygen donors, six of which are provided by the three ligand hydroxamate groups and two of which are provided by solvent molecules. To date, no crystal structure of this complex has been reported, yet the described coordination environment is consistent with the hexadentate nature of DFO observed in its Fe^3+^ complex [6] and with the results of computational studies on the Zr^4+^–DFO complex [7,8,9].

A pertinent model for such an octa-coordinated environment of Zr^4+^ is provided by the crystal structure of the Zr(Me-AHA)_4_ (Me-AHA = *N*-methyl acetohydroxamate) complex, which shows the metal ion surrounded by four hydroxamate groups [10]. Tetrachelate complexes of similar ligands, characterized by high thermodynamic stability, are also formed in aqueous solutions [11].

We have recently reported that, according to the evidences that we obtained through potentiometric and SAXS measurements in solution and by means of MALDI mass spectrometry, the Zr^4+^*–*DFO complex system is not only composed of 1:1 metal-to-ligand (M:L) complexes, as was generally believed, but the main species formed in a large pH region are characterized by larger aggregations with 2:2 and 2:3 stoichiometries. The stability constants of these complexes were obtained through the analysis of potentiometric measurements [8]. Interestingly, as early as 1992, W.E. Meijs et al. found that some UV spectral evidences they obtained with an excess of DFO could be explained by assuming that Zr^4+^ interacts with the hydroxamate groups of two different DFO molecules [12], which is in agreement with the formation of the 2:3 M:L complexes found by us. Nevertheless, another recent study by Toporivska et al., which was performed by adopting a mixed spectrophotometric/potentiometric approach based on the coordinative competition with Fe^3+^, did not find complex species with stoichiometries other than 1:1 [13]. In the same work, further analysis of the complexation equilibria at pH 1, which was performed by means of isothermal titration calorimetry, confirmed that the formed complexes contain Zr^4+^ and DFO in a 1:1 molar ratio, which is in agreement with both the 1:1 species reported in their work and the 2:2 complexes that are expected to form at pH 1 according to our equilibrium data [8].

To the best of our knowledge, when we thought about conducting the present study, no more information was available that could help settle this stoichiometry issue. We only knew that the ability of DFO to distribute its donor atoms between the coordination spheres of two metal ions in solution had also been documented for the complexation of other metal ions such as Cu^2+^ [14], Pb^2+^, Sn^2+^ [15], and Fe^3+^ [16]. Nevertheless, when this paper was almost ready to be submitted for publication, a paper by Imura et al. appeared in which the Zr^4+^/DFO system was reinvestigated by means of potentiometric measurements, and species of 1:1, 1:2, and 2:3 M:L stoichiometry were found to be the fundamental components of the complex system [17]. Very high stability constants were determined for these complexes. For instance, a logβ = 49.1 was determined for the ZrHDFO species, which exceeds the values obtained by Toporivska et al. [14] and by us (see ESI of [8]) by 2–4 orders of magnitude. Interestingly, the complexation model introduced by Imura et al. coincides with ours for the formation of polynuclear [Zr_2_H_5_(DFO)_3_]^4+^ and [Zr_2_H_6_(DFO)_3_]^5+^ complexes, in addition to mononuclear ones, but differs in terms of the formation of 1:2 instead of 2:2 species. These new data, although not available when we planned our work, increase the interest in the stoichiometry issue of Zr^4+^ complexes with DFO.

Recently, it was reported that the terminal amino group of DFO can be easily functionalized with an amino-3,4-dihydro-3-methyl-2-methoxy-5-nitroso-4-oxopyrimidine (Pm) group to form a deeply coloured (violet) ligand, hereinafter referred to as DFO*–*Pm (Figure 1), which is characterized by pH-dependent UV-vis absorption properties [18]. Both the deprotonation of the amine group bearing the pyrimidine substituent, which occurs in alkaline solution, and the protonation of the pyrimidine nitroso group, which occurs in acidic solution, give rise to marked changes in the adsorption spectra. Furthermore, DFO–Pm was shown to form very stable complexes with Cu^2+^ [18]. Other polydentate ligands that were functionalized with this pyrimidine group have been shown to form stable complexes with various metal ions. For all these complexes [18,19,20,21,22,23,24,25] as well as for DFO–Pm [18], metal complexation is manifested by evident modifications in the UV-vis adsorption spectra of the ligand.

This property suggests the possibility of exploiting the chromophoric characteristics of the pyrimidine group of DFO–Pm as an alternative/parallel method to potentiometry that could be used to follow the coordination of DFO to Zr^4+^ and to obtain additional information on the stoichiometry of the complexes formed. Indeed, as reported below, DFO*–*Pm is spectrophotometrically sensitive to Zr^4+^ coordination and furnishes clear stoichiometric information which, along with the equilibrium data from the potentiometric titrations and mass spectrometry results, reveals the formation of Zr^4+^ complexes with 1:1, 2:2, and 2:3 M:L stoichiometries.

Here, we report the results of this study, which were integrated with the analysis of the complexes formed by DFO–Pm with specific divalent and monovalent metal ions to better define the coordination properties of this ligand in relation to DFO.

## 2. Results and Discussion

### 2.1. Protonation/Deprotonation Equilibria of DFO–Pm

Protonation equilibria involving DFO–Pm in aqueous solution were previously studied under experimental conditions (0.1 M KCl, 298.15 K) [18] that were similar to those adopted in the present work (0.1 M Me_4_NCl, 298.15 K), and the results here obtained are in reasonable agreement. The determined protonation constants are listed in Table 1. Similar to DFO [26], DFO–Pm is also involved in four protonation equilibria occurring in the alkaline region (Figure S1 in Supplementary Materials), but the latter, unlike DFO, undergoes a fifth protonation in acidic media (pH < 4).

In agreement with other ligands containing the same pyrimidine group, this protonation equilibrium (corresponding to log*K* = 2.67 in Table 1) involves the -NO group of the pyrimidine, as confirmed by the variation of the UV-vis absorption spectra occurring below pH 4 (Figure S2). Also the pH dependence of the UV-vis absorption spectra of DFO*–*Pm had been studied in a previous work, with their behaviour being generally consistent with the spectra that we redetermined under our experimental conditions and are shown in Figure S2. Another difference between DFO–Pm and DFO, which is again attributable to the pyrimidine group of the former, concerns the first protonation stage, which takes place on the ligand’s primary amine nitrogen in the case of DFO, while in the case of DFO–Pm, it involves the same nitrogen atom that is now linked to the pyrimidine group and that is deprotonated. As this protonation equilibrium involves the chromophore (the pyrimidine), the absorption spectra signal its occurrence (Figure S2). The remaining three protonation stages involve the hydroxamate groups. The whole protonation sequence was confirmed by the pH-dependence of the ^13^C NMR signals recorded for DFO–Pm solutions [18].

### 2.2. Formation of Metal Complexes

To have a broader view of the coordinative properties of DFO–Pm compared to those of DFO, we studied the complexation equilibria involving DFO–Pm and several metal ions, such as Cu^2+^, Zn^2+^, Mg^2+^, Ca^2+^, Na^+^, and K^+^, in addition to Zr^4+^. The complex species formed and the corresponding stability constants were determined by computer analysis of the potentiometric titration data (0.1 M Me_4_NCl, 298.15 K), performed with the Hyperquad [27] program, are reported in Table 1. Despite DFO complexes with more than 20 different metal ions have been characterized in terms of their chemical speciation in solution, the alkaline metal cations appear to have been forgotten [26]. To partially remedy this shortcoming, we also turned our interests toward the complexation equilibria of DFO with Na^+^ and K^+^ and determined the corresponding stability constants, which have also been included in Table 1.

**Figure 2:**
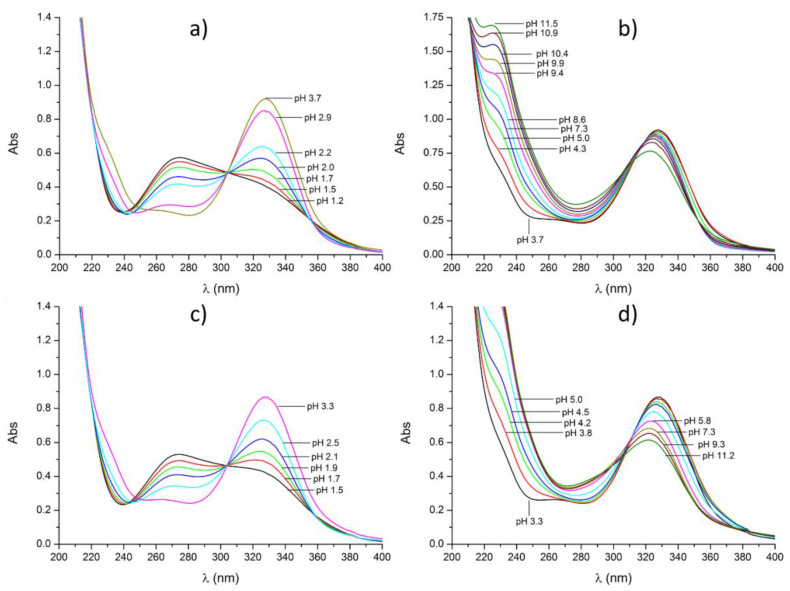
UV absorption spectra of solutions containing Cu^2+^ and DFO*–*Pm in 1:1 (**a**,**b**) and 2:1 (**c**,**d**) molar ratios at different pH values. (**a**) pH 1,2–3.7; (**b**) pH 3.7–11.5; (**c**) pH 1.5–3.3; (**d**) 3.8–11.2. [DFO*–*Pm] = 4.25 × 10^−5^ M, [Cu^2+^] = [DFO*–*Pm] in (**a**) and (**b**), [Cu^2+^] = 2[DFO*–*Pm] in (**c**,**d**).

The stability constants of the DFO complexes have been determined by different authors under relatively different temperature and ionic strength conditions. These constants were recently collected in a review paper that offers a rapid and clear overview of this ligand’s coordination properties [26]. A comparative analysis of the values that are available in the literature and of those determined in the present work (Table 1) for DFO and DFO–Pm allows us to draw some general conclusions. First, the two ligands give rise to metal complexes with very similar stoichiometries. This also applies to Zr^4+^ if the chemical speciation of DFO complexes that was previously reported by us is considered [8], while obvious discrepancies exist with the results of other studies, already mentioned, supporting complex systems that consist of only species with Zr^4+^:DFO 1:1 stoichiometry [13] or that include 1:2 instead of 2:2 species [17]. We will deal with this topic in more detail in a separate section. In the case of DFO–Pm, one more complex species can be formed in acidic solution where the ligand undergoes protonation on the pyrimidine. For Cu^2+^, it was not possible to verify the formation of binuclear complexes, which are formed by DFO, by means of potentiometric measurements due to precipitation occurring in solutions that contain Cu^2+^ and DFO–Pm in molar ratios equal to or greater than 1:1 at concentrations required by this technique. Nevertheless, at concentrations suitable for UV-vis measurements (4.25 × 10^−5^ M), all species are soluble and the formation of Cu^2+^ complexes with 2:1 M:L stoichiometry was proved. Indeed, upon the addition of Cu^2+^ to a solution of DFO–Pm at pH 6, a progressive spectral change was observed until a 2:1 M:L molar ratio was reached, but no further modifications were produced with further addition of the metal ion (Figure S3). As shown in Figure 2a,c, in the acidic region below pH 3.7, solutions with Cu^2+^:DFO–Pm 1:1 and 2:1 molar ratios show the same spectral variation with pH, while significant differences are found at higher pH (Figure 2b,d). The spectral variation occurring below pH 3.7 is very similar to that observed for the non-complexed ligand (Figure S2a): indeed, according to the stability constants in Table 1 (see also the species distribution diagram in Figure S4), complexation is almost non-existent at this pH and dilution (4.25 × 10^−5^ M) in the 1:1 Cu^2+^:DFO–Pm system. By analogy, this spectral behaviour suggests that significant complexation does not occur in the 2:1 system either. At higher pH, the spectra of 1:1 and 2:1 M:L solutions are different from the spectra of the free ligand, signalling that complexation takes place. A comparison between the pH dependence of selected absorbances (225, 274 and 328 nm) of the 1:1 and 2:1 M:L complexes is shown in Figure S5.

The spectroscopic response of DFO–Pm upon interaction with Zn^2+^ is shown in Figure S6.

Regarding the stability of the complexes, the completely deprotonated DFO–Pm ligand forms complexes that are moderately more stable than those formed by the completely deprotonated DFO, in agreement with the greater negative charge of the former (−4 for DFO*–*Pm versus −3 for DFO). The difference in complex stability becomes smaller if the form of DFO–Pm that only contains deprotonated hydroxamate groups, as in the case of DFO, is considered.

Accordingly, comparing the stability of M(II)HDFO*–*Pm (3 hydroxamate groups, ligand charge −3) with literature values [26] for M(II)DFO stability (3 hydroxamate groups, ligand charge −3) leads to excellent agreement (generally within 1 log*K* unit in different ionic media). Furthermore, it is important to note that the stability constants of the DFO complexes were determined in ionic media containing Na^+^ or K^+^ salts [26], metal ions that we have demonstrated to form weak complexes with this ligand. This means that some competition occurred in the binding of the studied metal ions, and consequently the determined stability constants resulted somewhat underestimated with respect to the stability constants determined in ionic media not containing Na^+^ or K^+^, such as in our measurements which were performed in 0.1 M Me_4_NCl. Accordingly, the stability differences observed between the DFO–Pm and DFO complexes would be even smaller if determined in the same ionic medium. In agreement with these considerations, the stability constants of the Zr^4+^ complexes with DFO–Pm that is not deprotonated at the amine group (log*K* = 35.97(8), Table 1) and with completely deprotonated DFO (log*K* = 36.14(9) [8]), which were determined in the same ionic medium (0.1 M Me_4_NCl), are equal within their experimental errors.

As a concluding note for this section, we can confidently say that DFO–Pm and DFO show similar coordination properties in terms of stoichiometry and stability of the formed complexes, at most with the exclusion of the very acidic and very alkaline regions in which DFO–Pm can generate additional species. For this reason, we believe that the chromophoric ligand DFO–Pm can be reasonably used as a model for DFO, in terms of coordination properties, and its photochemical sensitivity to metal coordination can be exploited to acquire additional information on the species formed. The pyrimidine chromophore that was appended to DFO to generate DFO–Pm is potentially a chelating group toward metal ions, and its involvement in metal coordination has been observed both in the solid state and in solution [28]. Nevertheless, we have no evidence of this group directly interacting with the metal ions in the equilibria studied here.

### 2.3. On the Stoichiometry of Zr^4+^ Complexes

DFO–Pm and DFO give rise to very similar complexation models with Zr^4+^ that contain 1:1, 2:2, and 2:3 M:L complexes. The few differences between these complexes are limited to the number of acidic protons that are involved in the complexation equilibria and can largely be ascribed to the fact that, with respect to DFO, DFO–Pm can undergo an additional deprotonation and an additional protonation in alkaline and acidic media, respectively. The stoichiometry of these complexes was determined by means of a computer-assisted analysis of the potentiometric (pH-metric) titrations, a method which, regardless of how powerful it is, is not exempt from possible misinterpretations, especially when the systems that are analysed are not simple, such as the one that is produced by DFO*–*Pm in the presence of Zr^4+^. For this reason, we have sought and obtained confirmation of the chemical speciation performed by potentiometry by means of spectrophotometric and mass spectrometry measurements.

**Figure 3:**
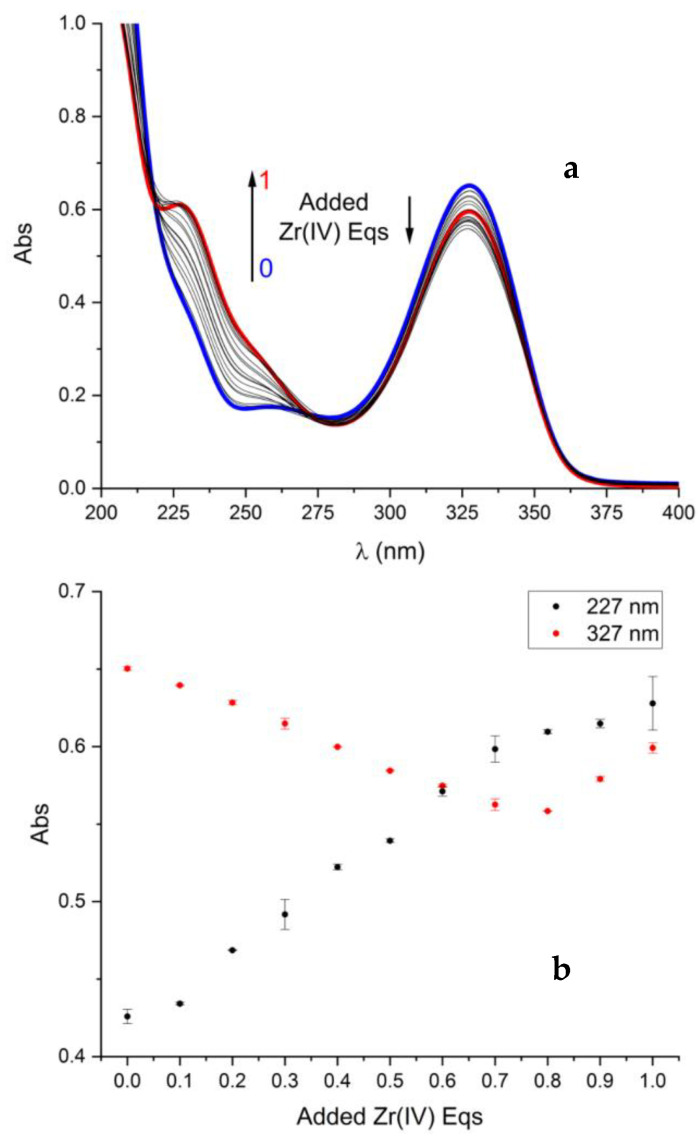
(**a**) UV absorption spectra of DFO–Pm solutions (3 × 10^−5^ M, pH 6) upon the addition of increasing amounts of Zr^4+^. (**b**) Variation of the absorptions at 227 nm and 327 nm with added Zr^4+^ equivalents (two replicate experiments).

A chromophoric pyrimidine group was bound to DFO to make it photochemically sensitive to metal coordination, and, as seen above for Cu^2+^ and Zn^2+^ coordination, this method is effective. It also works with Zr^4+^, a finding that will be discussed in more detail below. According to the results of our potentiometric measurements, it should be possible to demonstrate the formation of 2:3 species For instance, simulations performed with the Hyss [29] program show that additions of Zr^4+^ to a solution of DFO-Pm (3 × 10^−5^ M), from 0 to 1 equivalent, at pH 6, involve almost exclusively the uncharged form of DFO-Pm (H_4_L), and the 1:1 [ZrHL]^+^ and 2:3 [Zr_2_H_6_L_3_]^2+^ complexes, whose relative abundance changes with the M:L molar ratio along the titration. The results of two similar replicate experiments are shown in Figure 3a.

**Figure 4:**
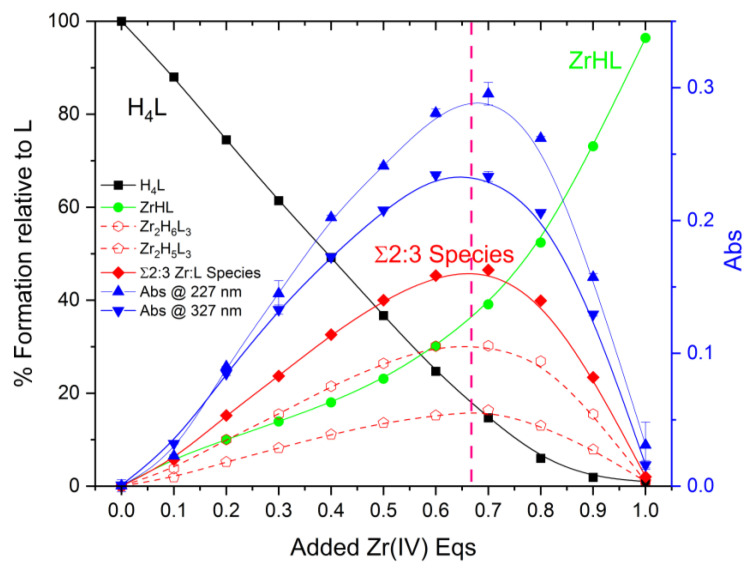
Variation in the absorption peaks at 227 nm (blue, ▲) and 327 nm (blue, ▼) with equivalents of added Zr^4+^, corrected for the contributions coming from H_4_L and [ZrHL]^+^. Two replicate experiments. [DFO*–*Pm] = 3 × 10^−5^ M, pH 6. Pink line represents theoretical 2:3 stoichiometric ratio.

As seen in Figure 3b, the variation in the absorbances at 227 nm and 327 nm shows a break at about 0.75 equivalents of added Zr^4+^, which is indicative of the formation of complex species with molar ratios that are smaller than 1:1. When these absorbances are corrected for the contributions from H_4_L and [ZrHL]^+^ (limiting spectra in Figure 3a), the molar ratio curves change shape, and define peaks centered at 0.64–0.68 (mean value 0.66) equivalents (blue curves in Figure 4) corresponding to the formation of species with 2:3 M:L stoichiometry, in agreement with the potentiometric results. It deserves to be noted that in the case of Zn^2+^, which only forms 1:1 complexes, the UV-vis spectra that are recorded upon the addition of increasing amounts of metal ion to DFO–Pm solutions do not result in any break in the spectral variation, as shown in Figure S7 for the 327 nm absorbance.

**Figure 5:**
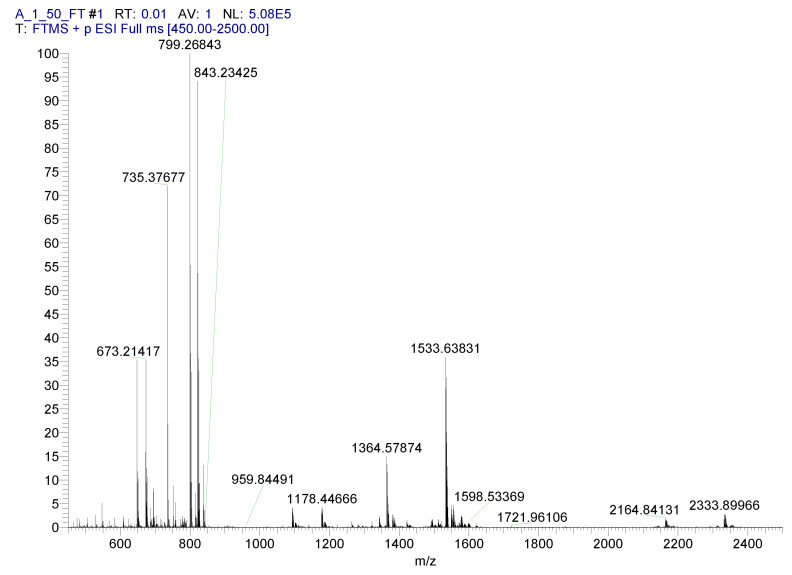
HRMS (ESI^+^) of 1:0.66 DFO*–*Pm:Zr^4+^ solution displaying 1:1, 1:2, and 2:3 M:L stoichiometries.

HRMS (ESI^+^) spectra experiments were performed under the conditions (pH, concentrations, M:L ratio) that, according to the potentiometric model, appeared to be most promising for the detection of species with stoichiometries other than 1:1. These conditions specifically targeted 2:3 species, whose abundance is sensitive to stoichiometric ratios. Figure 5 shows the high-resolution HRMS (ESI^+^) spectra that were obtained according to these conditions.

In broad terms, the spectrum is composed of the following groups of signals (approximate *m*/*z* in brackets): 2:3 Zr:L species (2330 and 2160 *m*/*z*), 1:2 Zr:L species (1530 and 1360), 1:1 Zr:L species (799 and 821), and Zr-free L (735). Table 2 lists the full assignments. This provides two pieces of information. First, non 1:1 Zr:L complexes are unambiguously formed and demonstrate faithful assignments for all species within a few ppm of the theoretical *m*/*z* values. Additionally, species containing 0, 1, or 2 Zr ions effectively display a markedly different fingerprint, which is expected due to the dominating isotopic distribution of Zr (H, C, N, O do not contribute much) (cf. Figure S8) [8]. This piece of evidence is direct experimental proof of the existence of such species and is independent from any modelling that is connected to indirect investigation methods (e.g., potentiometry). Second, there are some discrepancies between the expected species abundancies in solution and the spectral signals: not only are 1:1 and free L species far more abundant than expected, but 1:2 Zr:L species, which do not appear in our potentiometric model, are also encountered.

To determine whether the ESI MS conditions tamper with our sample, MS-MS spectra (Figure S9) were recorded by selecting the 2:3 species (peak at 2331 *m*/*z*) and applying to them a mild fragmentation procedure. Such experiments were extremely revealing because the 2:3 species were observed to break down into 1:2 (*m*/*z* 1533), 1:1 (*m*/*z* 821), and L species (*m*/*z* 735) (cf. Table 2), even at low collision energies. Additionally, the 1:2 species (peak at 1533 *m/z*) were shown to be able to breakdown into 1:1 and free ligand species (Figure S9).

When these data are put into perspective, the predominance of the 1:1 and 1:2 Zr:L species in HRMS (ESI), instead of the expected 2:3 species, suggests the dissociation of larger multinuclear complexes during this type of experiments. As this has now been proven for DFO–Pm, whose Zr^4+^ complexes are as stable or more stable (further possibility of deprotonation and extra negative charge) than DFO complexes (Table 1), a similar dissociation mechanism could also be envisaged for Zr^4+^:DFO complexes. In fact, both us and other research groups have initially only found 1:1 Zr^4+^:DFO complexes in ESI MS. While others have reported this information supporting the sole presence of 1:1 species [13], we have instead showed that with the MALDI/TOF technique, where ionization is milder and dilution is not required, stoichiometries other than 1:1 (namely 2:2 and 2:3) emerge [8].

This disagreement apparently prompted Imura et al. to re-perform MS experiments using different experimental methods. They indeed found 1:2 and 2:3 species using Nano-ESI-Q-MS, a finding that is in full agreement with the DFO*–*Pm MS data. This further demonstrates the sensitivity of the system to ionization processes. Imura and co-workers stated that since the existence of 1:2 species was suggested by the potentiometric data (whose validity is further discussed in a forthcoming section), two-fold ligand excess was used for the MS experiments. In such conditions (Figure 3 in [17]) and at the stated working pH 7.0, 1:2 and 2:3 species should be almost equally abundant. In the MS spectra (Figure 2 in [17]), however, 2:3 species are hardly found, while 1:2 species are dominant. This suggests again that the 2:3 species decompose into 1:1 and 1:2 ones during MS experiment, a phenomenon that we have demonstrated for DFO–Pm.

It is also interesting to notice how all the Zr^4+^–DFO–Pm species, regardless of their stoichiometries, are observed as Na^+^ complexes in MS spectra (cf. Table 2). The same was true (with K^+^, probably from glass electrode) for the previously studied non 1:1 Zr^4+^:DFO species [8]. In other words, the MS spectra testify the ability of DFO and DFO–Pm ligands (or clusters of them) to simultaneously interact with more than one metal cation (Zr^4+^ and Na^+^/K^+^) at the same time. This behaviour requires attention: in experiments that are based on competition between two different metal ions, the possible formation of heterodinuclear complexes (or larger aggregations) must be considered and excluded or evaluated on an experimental basis.

### 2.4. An Attempt to Arrive at an Unified Model for the Zr^4+^/DFO System

As previously mentioned, three conflicting speciation models have been reported for Zr^4+^/DFO [8,13,17]. This issue has also started to become fairly debated in the secondary literature [26,30]. At this point, we deemed it imperative to attempt to find a common ground between the three available models.

It can be anticipated that the results of this analysis show that the only element unifying these three speciation models is that they all crucially depend on the model adopted for metal hydrolysis. For this reason, we shall look at published data without any assumptions as to what the correct hydrolysis model for Zr^4+^ should be: each one is free to choose their own, provided that the experimental work is then performed in accordance with the chosen model. Therefore, the adopted models will be mentioned to inform readers but will not be discussed. Debate on Zr^4+^ hydrolysis and its modelling can be rightfully considered to be a very advanced topic that requires specialized knowledge of solution thermodynamics and first-hand experience in determining stability constants in order to be fully understood. This is beyond the scope of present study. This is especially the case because the puzzling discrepancies among the three DFO/Zr^4+^ speciation models can instead be elucidated by means of much more beginner-friendly thermodynamic considerations.

#### 2.4.1. Model by Imura et al.

The premise of the experimental work by Imura et al. [17] is setting the matter of Zr^4+^/DFO speciation building on the discrepancy between the model proposed by Toporivska et al. [13] and our own [8].

Apparently, these authors did not realize that one of the two models needed a critical revision (see below). Additionally, the great difference (about 10^10^), claimed by these authors, between the stability constants reported by Toporivska et al. [13] and by us [8] for ZrHDFO, which has aroused surprise and discussion in their work, is smaller and is the result of an error they made in assigning to ZrHDFO the stability constant that we reported for ZrDFO, an error of about nine orders of magnitude (log*K*_1,1_ = 36.14 for ZrDFO vs. logβ_1,1,1_ = 44.7 for ZrHDFO) [8].

When attempting an informed examination of data, a major issue, concerning the 1:2 species that they found and the conditions adopted to find those species in potentiometric titrations, arises. As previously mentioned, all studies on the matter have relied on the competition between DFO and OH^-^ in binding Zr^4+^. For the computer-aided processing of their measurements, Imura et al. adopted a set of hydrolysis constants (for not specified Zr(OH)*_s_* species) taken from a literature model that included 26 species, most of which were bi-, tri- and tetra-nuclear and whose hydrolysis constants were predicted by a computational approach based on a hard sphere model [31].

The use of the DFO/OH^−^ competition method is dictated by the fact that DFO protonation is not strong enough to compete with Zr^4+^ coordination, even at the lowest possible pHs that are accessible for potentiometric titrations. Indeed, in all potentiometric titrations performed on the Zr^4+^/DFO system [8,13,17] (as well as for Zr^4+^/DFO*–*Pm), Zr^4+^ complexes are 100% formed under the most acidic conditions adopted. Nevertheless, upon the addition of a strong base (OH^-^ anions), the formation of Zr^4+^ hydroxo complexes are strong enough to cause the decomposition of the DFO (or DFO–Pm) complexes. This is the criterion on which the competition method with OH^-^ anions is based. It appears quite evident that, in order to be effective, this method requires that the potentiometric titrations are performed in such a way that an extensive decomposition of the DFO complexes occurs upon addition of the OH^-^ titrant solution, in favour of an extensive formation of Zr^4+^ hydroxo complexes, safeguarding the requisite for all equilibrium determinations that both associated and non-associated species must be simultaneously present in measurable concentrations [32]. This does not occur in the measurements reported by Imura et al. A very modest formation of Zr^4+^ hydroxo complexes is observed for the titrations performed with 1:2 and 1:1.43 M:L ratios, while no competition at all occurs with OH^-^ anions (i.e., there is no formation of Zr^4+^ hydroxo-complexes) when the excess of ligand is increased to force the formation of 1:2 complexes (see Figure 3 in [17]).

We would also like to point out that the slim amount of competition existing in such experiments (Figure 3 in [17]) in alkaline medium is because ZrHDFO is the least protonated complex species included in the model. If a ZrDFO species was introduced, thus strengthening the ligand competition with OH^-^ ions even at more alkaline pHs, then there would likely be no competition observed in the experiments at all, totally overthrowing the proposed thermodynamic results. There are two reasons to expect that such a species must exist. First, the fourth deprotonation of metal-free DFO involves the extraction of a proton from the ammonium group of a linear chain containing three hydroxamate group, i.e., it is the deprotonation of a zwitterion with a total charge of −2 to give an anion a total charge of −3. The deprotonation of ZrHDFO is instead the deprotonation of a +2 cation to give a +1 one. According to simple electrostatic considerations, the first process, which does take place, is expected to be more disfavoured than the second one. Despite this fact, the first process was found to be active by Imura et al., and the second one was not. The second reason is that such species was found to be a representative one for several metals, including: Mg^2+^, Ca^2+^, Sr^2+^, Mn^2+^, Co^2+^, Ni^2+^, Cu^2+^, Zn^2+^, Pb^2+^, Cd^2+^, Sn^2+^, Fe^3+^, Al^3+^, Ga^3+^, In^3+^, Mn^3+^, Co^3+^, La^3+^, Yb^3+^, Bi^3+^, Th^4+^, Pu^4+^, and even Zr^4+^ [26], and this species was also found by Imura et al. in their ESI-Q-MS spectra.

In addition, the values of the stability constants of the complexes reported by Imura et al. also generate some perplexity. Let us consider, for example, the stability constants of the ZrH_X_(DFO)_2_ species (x = 2–5) [17] and rewrite their formation equilibria Zr + xH + 2DFO = ZrH_X_(DFO)_2_ in the form ZrHDFO + H_X−1_DFO = ZrH_X_(DFO)_2_, which corresponds to the addition of a H_X−1_DFO ligand (i.e., DFO in different protonation states) to the already formed ZrHDFO complex (charges omitted for simplicity). Electrostatic, probabilistic, and basic thermodynamic considerations would predict the following: the addition of a fully protonated H_4_DFO^+^ species (ligand with no free hydroxamates and positively charged) to ZrHDFO^2+^ to afford ZrH_5_DFO_2_^3+^ should not be a relevant process; the addition of H_3_DFO (one free hydroxamate group, uncharged ligand) should happen with a significantly higher log*K* with respect to the above; the addition of H_2_DFO^−^ (two free hydroxamate groups, negatively charged ligand) or HDFO^2−^ (three free hydroxamate groups, more negatively charged ligand) to the ZrHDFO^2+^ complex should happen with progressively increasing log*K* values. The values that were obtained by Imura et al. [17] were instead log*K*/free hydroxamate groups on the entering ligand = 5.89/0, 6.27/1, 5.83/2, 5.69/3. Not only these data do not follow a reasonable trend, but they also sustain that the addition of a fully protonated ligand, unable to bind, to an already formed strong complex produces gigantic stabilization (about 6 log units). It should be noted that negligible stability is expected to be possibly gained by rearrangement of protonated and metalated sites in such complexes, as the differences in the protonation constants among hydroxamate groups in free DFO are limited to ≈1 log unit (all sites have very similar acid/base properties), i.e., five orders of magnitude less than the amount required to justify the claimed stability of ZrH_5_(DFO)_2_.

Based on the above reasons, we are not confident in the potentiometric results that were obtained by Imura et al.; however, we are comforted by the results of the mass spectrometry study they performed, which evidenced the presence of 1:1, 2:3, and 1:2 Zr^4+^:DFO complexes. Species with a 1:2 stoichiometric ratio, whose formation may have been favoured by the appropriate selection of the M:L ratio, were also found in the MS measurements of this work, and were generated by the fragmentation of 2:3 Zr^4+^:DFO–Pm complexes. Accordingly, it seems reasonable to expect that similar 1:2 complexes might also be formed in solution, under appropriate conditions, but the quantification of their formation (i.e., the determination of their stability constants) proposed by Imura et al. appears to be somewhat unreliable.

#### 2.4.2. Model by Toporivska et al.

Toporivska et al. [13] also based their model on potentiometric titrations and relied on the competition with OH^−^ ions; in the case of their study, appropriate competition seems to be taking place [13]. They arrived at the conclusion that only 1:1 Zr^4+^:DFO complexes are formed, and they then sought to verify their results by determining the stability constant of the ZrHDFO species by means of spectrophotometric measurements. For this purpose, three different spectrophotometric titrations were performed independently, which were based on the claimed competition that should occur at pH 2 between Zr^4+^ and Fe^3+^, a pH which, they say, should avoid the hydrolysis of metal ions and should prevent the decomposition of DFO. The three titrations were as follows: (a) 1:1 Fe^3+^: Zr^4+^ solution titrated with increasing equivalents of DFO (0 to 4 eqs), (b) 1:1 Fe^3+^:DFO solution titrated with Zr^4+^ (0 to 3 eqs), and (c) 1:1 Zr^4+^:DFO solution titrated with Fe^3+^ (0 to 300 eqs). Only the ZrHDFO complex was assumed to form, and the coloured species they followed were the Fe^3+^ complexes of DFO. The hydrolytic models they adopted for Zr^4+^ and Fe^3+^ were taken from the literature [33,34].

In the following, we will assume that the reported speciation is fully valid. The modelling predictions that we report below were made using the Hyss program [29] and with the speciation results and the experimental conditions that were reported in the original paper [13].

The spectrophotometric results from which Toporivska et al. calculated their stability constants for ZrHDFO are shown in Figure 3 of [13]. In experiment (a), DFO added to an Fe^3+^/Zr^4+^ 1:1 mixture, the modelling predicts that the end of the experiment is reached when two equivalents of DFO are added (Figure S10). The first equivalent selectively binds Zr^4+^ (no spectral change should occur), the second one binds Fe^3+^, and the other two equivalents of DFO remain free and should cause no spectral changes whatsoever. Yet, spectral changes are observed from 0 all the way up to 4 eqs [13]. Our predictions dictate that in such an experiment, there is no Fe^3+^/Zr^4+^ competition for DFO (the difference in stability is excessive); therefore, no binding constant can be determined in such conditions. The fact that spectral changes are observed at DFO eqs < 1 and > 2 indicate that further processes might be active and undermines the assumption that only 1:1 species are present, which is the foundation of the whole experimental design. On one hand, the changes observed at DFO eqs < 1. denoting that Fe^3+^ is coordinated from the beginning of the titration, might be indicative of the formation of heteropolynuclear Fe^3+^/Zr^4+^ DFO complexes. Otherwise, taking into account that kinetic problems have been excluded by the authors, the only alternative explanation is that the stability difference between the Fe^3+^ and Zr^4+^ complexes must be much smaller than that reported. On the other hand, spectral changes at DFO eqs > 2 suggest the formation of complexes with M:L ratios that are smaller than 1:1.

In experiment (b), Fe^3+^-DFO titrated with increasing amounts of Zr^4+^, three equivalents of Zr^4+^ are necessary to completely flatten the Fe^3+^-DFO band. Modelling predictions show that the first Zr^4+^ eq should displace Fe^3+^ quantitatively (Figure S11); this is because, again, the claimed Fe^3+^/Zr^4+^ competition for DFO does not exist: Zr^4+^ quantitatively extracts the ligand from the Fe^3+^ complex. Again, no binding constants can be determined from this kind of experiment. The fact that the large LMCT band centred at 430 nm, characteristic of trihydroxamate Fe^3+^ species, decreased gradually upon the addition of up to 3 eqs of Zr^4+^ suggests again the formation of complexes with M:L ratios different from 1:1 or a smaller difference between the stability of the two competing complexes.

Experiment (c), 1:1 Zr^4+^:DFO solution titrated with Fe^3+^ (0 to 300 eqs), is interesting and revealing (Figure S12). Competition indeed exists in this case. Modelling simulations show that this is because of further equilibria: should one neglect hydrolysis, no competition would exist in the system, as above, i.e., Fe^3+^ does not compete with Zr^4+^ for DFO (more than 10^4^ eqs of Fe^3+^ would be necessary to displace about 10% Zr^4+^ from the DFO complex in the absence of hydrolytic equilibria). The reason for competition to be there in the real experiment is the presence of hydrolysis: the only reason why Zr^4+^ is displaced by Fe^3+^ is because the former forms more stable hydroxo species under the experimental pH conditions. In other words, the whole experimental set up of spectrophotometric measurements, aimed at bypassing the hydrolysis problem, ends up performing only one meaningful experiment out of the three, and the only competition that exists in those experiments is entirely due to hydrolysis. It should also be mentioned that it takes a lot of faith to believe that the complexes would retain their 1:1 stoichiometry after the addition of a 300-fold excess of Fe^3+^ to DFO.

The fact that three measurements, two of which are not eligible for the determination of stability constants, all result in logβ values that are in good agreement is somewhat surprising. Their average logβ value of 47.6 is about one order of magnitude greater that the potentiometrically determined logβ value of 46.4 [13].

As a final note, when these authors tried to confirm their potentiometric results with ITC technique at pH 1, the discrepancy between their own two apparent stability constants was as large as a 10^6^ factor [13].

#### 2.4.3. Model by Savastano et al.

As previously mentioned, all potentiometric measurements conducted in the three studies are based on DFO/OH^−^ competition for Zr^4+^. In our case [8], the employed hydrolysis model for Zr^4+^, which is crucial, only consists of [Zr(OH)_n_]^(4 − n)+^ (*n* = 1–4) species, as it was determined by us under the same experimental conditions of the complexation measurements (potentiometric titrations). We have since faced some criticism for not involving polynuclear species in the hydrolysis model, in contrast with exhaustive studies by Ekberg et al. [33] (used as reference model in [13]). The reason for their exclusion is simple: their inclusion did not serve to fit our experimental curves and they were therefore discarded by the HYPERQUAD program [27] used to compute the equilibrium constants.

It should be noted that a comprehensive review by Rai et al. [35], which is almost contemporary to our studies, was dedicated to a critical re-review of the NEA (Nuclear Energy Agency) model (based on Brown et al. one [36]). This new study also proposes the elimination of polynuclear Zr^4+^ species from the hydrolysis model and its essential reduction to simple mononuclear hydroxospecies. Discrepancies among existing models can span several log*K* units: for the relevant Zr^4+^ + 4OH^−^ = ZrOH_4_(aq) equilibrium, Brown et al. recommend a log*K* of 53.81, while Rai et al. defined an upper bound log*K* < 45.89.

One may suspect that spurious species may have resulted from our calculations of the complex stability constants due to the hydrolysis model that we employed, specifically because of the absence of polynuclear hydroxo species, and that this might be the root of the polynuclear Zr:DFO species we found. In order to address these legitimate concerns, in the following section, we will re-evaluate our own work to account for alternative hydrolysis models.

A few considerations have to be made before discussing the data obtained from any alternative hydrolysis model. The experimental potentiometric curve for Zr–DFO complexation obviously does not depend on the chosen hydrolysis model. In all studies [8,13,17], competition is among the least protonated Zr–DFO species included in the model and Zr(OH)_4_. Almost no other hydrolysis products were formed during the titrations, regardless of the hydrolysis model chosen by the authors [8,13,17]. This introduces a crucial dependence of the calculated stability constants for the Zr–DFO complexes on the logβ values that is assigned to Zr(OH)_4_, while most other species, including polynuclear ones, tamper very little with the overall fitting, as they are not formed, regardless of the experimental conditions. That is, changing the stability of Zr(OH)_4_ changes the stability of the Zr–DFO species.

**Figure 6:**
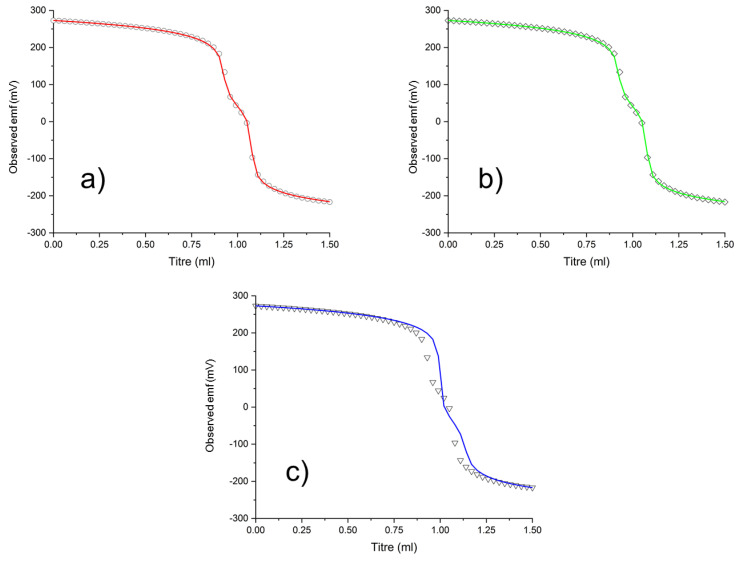
(**a**) Sample 1:1 DFO:Zr titration curve from our previous work [8]. Fitting of experimental points performed with our Zr^4+^ hydrolysis model results in the red calculated profile and logβ values in Column 1 of Table 3. (**b**) Fitting performed with the same hydrolysis model as ref. [13], allowing for 2:2 and 2:3 Zr:DFO species results in the green curve and logβ values in Column 3 of Table 3. (**c**) Fitting performed with the same hydrolysis model as in ref. [13], limiting stoichiometry to 1:1 Zr:DFO species only, affords blue curve and the data in Column 4 of Table 3.

Accordingly, we decided to fit our DFO data using the hydrolysis model by Ekberg et al. [33], which was employed in the work by Toporivska et al. [13] (Imura et al. unfortunately did not include their exact hydrolysis model [17]). The data can indeed be successfully refitted with the same species of 1:1, 2:2 and 2:3 stoichiometries, as shown in Table 3 and in Figure 6b. As Zr(OH)_4_ became more stable (about 5 log*K* units), the Zr–DFO complexes that were directly competing with it (ZrDFO and ZrDFO(OH)) were also found to be more stable (about 5 log*K* units). Global speciation, including 2:2 and 2:3 species, is only re-adjusted to account for such extra stability, but the “spacing” of the new logβ values (i.e., log*K* values of relevant processes for the system and its speciation) remain unchanged (cf. columns 5 and 6 of Table 3). Because of this choice, since a common logβ value is used for Zr(OH)_4_, the discrepancies with the constants obtained by Toporivska et al. [13], which were originally about 5 log*K* units, are virtually eliminated (less than 1 log*K* discrepancy on 101 species, Table 3), as it could have been anticipated.

Conversely, if we maintain the hydrolysis model that was used by Toporivska et al. [13] and we try to fit our potentiometric curves with Zr:DFO 1:1 species alone, then the experimental curves can no longer be reproduced by the model, as shown in Figure 6c, although stability constants of acceptable accuracy can still be calculated (Table 3, column 4).

In other words, a rather large pH region exists in which further species must be present, and this is likely not caused by the presence of polynuclear hydroxospecies, which are now included in the model. This problematic pH region is centered around neutral pH, where superable but relevant kinetic issues arise [8].

The only way to overcome this issue was to include 2:3 and 2:2 species in the model, regardless of the chosen hydrolysis model (changing logβ values for Zr(OH)_n_]^(4−n)+^ (*n* = 1–4) species changes the values of the stability constants of the DFO*–*Zr^4+^ complexes, but not the experimental curve profile). We are quite comforted by the fact that the potentiometric measurements by Imura et al. [17], who used a third independent hydrolysis model, also required 2:3 species to be fitted, moreover agreeing with us on their protonation state.

Since we did not originally succeed in demonstrating the existence of polynuclear species by ESI MS (Imura et al. succeeded in doing it, and we succeeded with DFO*–*Pm in present study), we tried MALDI TOF, where it was possible to show both 2:3 and 2:2 species. Since the 2:2 dimer is prone to dissociation upon dilution (e.g*.,* under common conditions for ESI, but nor for MALDI), we searched and found it in SAXS experiments, which require a significant concentration, directly in solution, and showed that the scattering objects in solution correspond to DFT prediction.

It is quite manifest that the main 1:1 and 2:3 stoichiometries are accompanied by other species whose abundance might depend on the experimental conditions. We took care in both the Zr^4+^/DFO and Zr^4+^/DFO*–*Pm systems to have effective DFO/OH^−^ competition, which meant keeping the excess ligand below or equal to what was needed for a 1:2 M:L ratio in our systems. Under similar conditions, 2:2 dimers are relevant, but we did not find 1:2 species with our potentiometric measurements. Instead, these were observed in the MS experiments of Imura et al. as well as in ours. We cannot exclude that such species can be formed in the presence of a greater excess of ligand.

In brief, lack of convergence on Zr–DFO exact stability is due to the lack of a single unambiguous hydrolysis model. Yet, regardless of the Zr(IV) hydrolysis model (mononuclear/polynuclear species) that is used, a 1:1 Zr:DFO model is unable to reproduce experimental data. This demonstrates that non-1:1 complexes are not spurious species resulting from an incorrect hydrolysis model. If they were, they could not be found by other non-potentiometric methods, which is contrary to the evidence that we generated using multiple techniques (ESI MS, MALDI TOF, solution SAXS, DFT, now HRMS and UV-Vis with DFO*–*Pm) and to the evidence provided by Imura et al. [17].

#### 2.4.4. A Unified Model for Zr(IV):DFO System?

Not yet. In analyzing the three proposed models, we have strengthened our belief that the system is far from the 1:1 only simple case. Among available models, ours remains robust and reliable: serious critical issues have been identified in the definition processes of the other two. Even so, our model remains dependent on Zr^4+^ hydrolysis. Its main flaw, sensitivity towards alternative hydrolysis models, has been investigated, and the robustness of the model has been demonstrated. It is understood that our model can and will be subject to recalculation and updates as soon as broader agreement is reached on a suitable model for Zr^4+^ hydrolysis.

In this context, any additional meaningful information that can be derived from well-thought experiments would be valuable for ascertaining speciation in such a challenging system.

## 3. Materials and Methods

### 3.1. Materials

All reagents were purchased from commercial sources and were used as received. Solvents were of analytical grade and were used without further purification. Water used for potentiometric and spectroscopic measurements was twice distilled and passed through a Millipore apparatus (Millipore, Burlington, MA, USA). Commercial Me_4_NCl used as the background electrolyte was recrystallized from isopropyl alcohol and dried under vacuum to constant weight. The ligand DFO–Pm was synthetized according to a reported procedure [18]. The only modification that was introduced was the usage of excess solid K_2_CO_3_ instead of KOH to neutralize the DFO*–*mesylate salt, with excess carbonate being conveniently discarded in the same filtration step required to remove excess pyrimidine reagent.

### 3.2. Potentiometric Measurements

Potentiometric (pH-metric) titrations, for the determination of the equilibrium constants were performed in 0.1 M Me_4_NCl at 298.1 ± 0.1 K using an automated apparatus and a procedure that has been previously described [11,37]. The acquisition of the emf data was performed with the computer program PASAT [38,39]. A combined electrode (Metrohm 6.0262.100, Metrohm, Herisau, Switzerland) was calibrated as a hydrogen-ion concentration probe through the titration of standardized HCl solutions with standardized CO_2_-free NaOH solutions and by determining the equivalent point using Gran’s method [40], which furnishes the standard potential (E°) and the ionic product of water (pK_w_ = 13.83(1) in 0.1 M Me_4_NCl at 298.1 K). The HYPERQUAD [27] computer program was employed to calculate the stability constants from potentiometric data. The concentration of the ligands [L] was about 5 × 10^−4^ M in all of experiments, while the concentrations of the metal ions were in the ranges of 0.5–1.1 [L] for Zr^4+^; 0.5–0.8[L] for Zn^2+^, Cu^2+^, Ca^2+^, and Mg^2+^; and 5–10[L] for Na^+^ and K^+^. In the case of Zn^2+^ and Cu^2+^, complex precipitation was observed for metal concentrations that were equal to or greater than ligand concentration. In the case of Ca^2+^ and Mg^2+^, the metal concentration was kept lower than ligand concentration to avoid the formation of metal hydroxide in the alkaline solutions. The studied pH range was 2.0–11.2.

At least two measurements were performed to determine ligand protonation and the metal complexation constants (three for Cu^2+^ and four for Zr^4+^ complexes). The behaviour of DFO*–*Pm and the experimental attention required to reach equilibrium were very similar to what had previously been observed for DFO [8].

### 3.3. Spectrophotometric Measurements

Absorption spectra were recorded at 298 K on a Jasco V-670 spectrophotometer (Jasco Europe, Lecco, Italy). For the UV-vis spectra, the ligand concentration was 4.25 × 10^−5^ M for the measurements with Cu^2+^ (4.25 × 10^−5^ M or 8.50 × 10^−5^ M) and 3 × 10^−5^ M for the measurements with Zn^2+^ and Zr^4+^, with the concentration of the metal ions ranging from 3 × 10^−6^ M to 3 × 10^−5^ M.

### 3.4. Mass Spectrometry

A 1:0.66 L: Zr^4+^ solution ([L] = 1 mM) at pH 4.0 (>90% formation of the Zr_2_H_6_L_3_ according to potentiometric data) was prepared in LC-MS grade water and was further diluted 50 times prior to injection with the same solvent (final pH 5.5, ≈ 70% formation of the Zr_2_H_6_L_3,_ 15% ZrHL, and 10% H_4_L according to potentiometric data). ESI-HRMS spectra were recorded by directly introducing the samples at a flow rate of 10 μL/min in an Orbitrap high-resolution mass spectrometer (Thermo, San Jose, CA, USA). The instrument was calibrated just before the analyses (external calibration). The working conditions were the following: positive polarity, spray voltage 5 kV, capillary voltage 50 V, capillary temperature 270 °C, tube lens voltage 100 V. The sheath and the auxiliary gases were set at 10 (arbitrary units) and 2 (arbitrary units), respectively. Xcalibur 2.0. software (Thermo, San Jose, CA, USA) was used for spectra acquisition, and a nominal resolution (at *m*/*z* 400) of 100,000 was used. The experimental parameters for the MS/MS experiments were as follows: *m*/*z* 2331: CE (collision energy) 15, MS (isolation width) 3, elapsed scan time (sec) 3.10, ACT.Q (activation *q* value) 0.250; *m*/*z* 1536: CE (collision energy) 25, MS (isolation width) 3, elapsed scan time (sec) 2.64, ACT.Q (activation q value) 0.250.

## 4. Conclusions

A simple DFO modification, performed by adding a chromophoric group to its terminal amine function, provided a ligand with coordination characteristics very similar to those of the pristine molecule but that were enriched with the further property of being able to spectroscopically signal its interaction with metal ions in solution. Indeed, the new ligand, called DFO*–*Pm, provided a spectrophotometric response upon the coordination of Cu^2+^, Zn^2+^, and Zr^4+^. This spectrophotometric response was used to confirm the stoichiometry of the formed complexes. Accordingly, the UV-vis spectral data along with potentiometric results supported a complexation model for the Zr^4+^/DFO*–*Pm system composed of variously protonated 1:1, 2:2, and 2:3 M:L complexes, in strong agreement with results previously obtained by us [8] for DFO, both in terms of the stoichiometry of complexes and in terms of their stability. The ESI MS spectra, addressed to identify the 2:3 M:L complexes, provided evidence for the formation of these species as well as of 1:1 and 1:2 ones, the latter of which had never been observed in our potentiometric studies, neither now with DFO*–*Pm nor before with DFO [8]. Further mass spectrometry investigations performed under mild fragmentation conditions revealed that the 1:2 species are generated, along with 1:1 and Zr^4+^-free ligand ones, by fragmentation of 2:3 complexes, even at low collision energies.

The results obtained with DFO*–*Pm reinforce our confidence in our previously reported [8] complexation model for the Zr*–*DFO system. After a careful analysis of the other two models available, we believe that our speciation model is the most reliable so far, the other two having been determined either under experimental conditions that do not comply with the necessary requirements for a correct analysis of chemical equilibria or disregarding the experimental evidence indicating that species with a stoichiometry other than 1:1 M:L are also formed.

Indeed, it seems clear from the results of the three models discussed here [8,13,17], including the one by Toporivska et al. [13], which aims to demonstrate the formation of only 1:1 Zr^4+^:DFO complexes but yields evidence for complexes of different stoichiometries, and from those herewith presented for the DFO*–*Pm model ligand, that aggregations larger than 1:1 M:L, including 2:2, 2:3 and, tentatively, even 1:2 complexes, are needed to justify the entire body of experimental information available for the Zr^4+^/DFO system. These larger aggregations are not marginal but may be prevalent species depending on the experimental conditions (pH, M:L ratio).

The ability of DFO/DFO*–*Pm to form both homodinuclear (Zr^4+^/Zr^4+^) and heterodinuclear (Zr^4+^/Na^+^ and Zr^4+^/K^+^) complexes, shown by potentiometry and MS results, opens doubts on the use of competition with other metals to determine the stability constants of Zr^4+^ complexes with similar highly dentate ligands; it does not deny that this method is applicable, but it suggests that the heteronuclear system should be studied in detail and that a priori assumptions about the independence of the two separate metal systems should be avoided.

It seems also clear that all three models presently available for the Zr^4+^/DFO system crucially depend on the model adopted for metal hydrolysis. Several hydrolysis models have been proposed for Zr^4+^ [8,31,33,41,42,43,44,45,46,47], among which one can choose the one he deems the most appropriated for his case, but the fairest choice is that everyone analyses his own model under the exact experimental conditions he will use for the complexation reactions, even though this is notoriously a demanding task.

From the above, there is no doubt that Zr^4+^/DFO is a challenging system. Nonetheless, given its applicative and academic interest, it would be desirable that other research groups with proven experience in determining stability constants contribute to verifying the data that are currently available or offer competent alternative solutions.

## Figures and Tables

**Table 1:** Ligand protonation constants and stability constants of metal complexes with DFO–Pm (H_4_L) determined in 0.1 M Me_4_NCl at 298.15 K. Stability constants of the complexes formed by DFO (H_3_DFO) with Na^+^ and K^+^ under the same experimental conditions are also included. Values in parentheses are standard deviations on the last significant figures.

Equilibria	log*K*	Equilibria	log*K*
L^4−^ + H^+^ = HL^3−^	10.70(2)	H_3_L^−^ + Zn^2+^ = [ZnH_3_L]^+^	5.4(1)
HL^3−^+ H^+^ = H_2_L^2−^	9.53(3)	H_4_L + Zn^2+^ = [ZnH_4_L]^2+^	3.43(3)
H_2_L^2−^ + H^+^ = H_3_L^−^	9.07(3)		
H_3_L^−^ + H^+^ = H_4_L	8.40(3)	L^4−^ + Cu^2+^ = [CuL]^2−^	15.44(8)
H_4_L + H^+^ = H_5_L^+^	2.67(3)	HL^3−^ + Cu^2+^ = [CuHL]^−^	14.29(9)
		H_2_L^2−^ + Cu^2+^ = [CuH_2_L]	13.49(7)
L^4−^ + Na^+^ = [NaL]^3−^	2.35(5)	H_3_L^−^ + Cu^2+^ = [CuH_3_L]^+^	8.89(3)
HL^3−^ + Na^+^ = [NaHL]^2−^	1.6(1)		
		L^4−^ + Zr^4+^ = [ZrL]	37.40(5)
L^4−^ + K^+^ = [KL]^3−^	2.11(7)	HL^3−^ + Zr^4+^ = [ZrHL]^+^	35.97(8)
		L^4−^ + Zr^4+^ + 2H^+^ = [ZrH_2_L]^2+^	51.51(8)
L^4−^ + Mg^2+^ = [MgL]^2−^	4.42(3)	2L^4−^ + 2Zr^4+^ + H^+^ = [Zr_2_HL_2_]^+^	88.63(5)
HL^3−^ + Mg^2+^ = [MgHL]^−^	4.30(3)	2L^4−^ + 2Zr^4+^ + 3H^+^ = [Zr_2_H_3_L_2_]^3+^	102.3(1)
H_2_L^2−^ + Mg^2+^ = [MgH_2_L]	3.16(8)	3L^4−^ + 2Zr^4+^ + 4H^+^ = [Zr_2_H_4_L_3_]	125.12(6)
		3L^4−^ + 2Zr^4+^ + 5H^+^ = [Zr_2_H_5_L_3_]^+^	134.0(2)
L^4−^ + Ca^2+^ =[CaL]^2−^	3.82(6)	3L^4−^+2Zr^4+^+6H^+^=[Zr_2_H_6_L_3_]^2+^	142.4(2)
HL^3−^ + Ca^2+^=[CaHL]^−^	3.35(7)		
		DFO^3−^ + Na^+^ = [NaDFO]^2−^	1.70(9)
L^4−^ + Zn^2+^ = [ZnL]^2−^	10.88(5)		
HL^3−^ + Zn^2+^ = [ZnHL]^−^	10.54(4)	DFO^3−^ + K^+^ = [KDFO]^2−^	1.57(7)
H_2_L^2−^ + Zn^2+^ = [ZnH_2_L]	8.62(3)		

**Table 2:** HRMS (ESI+) spectrum assignment.

Stoichio-Metry	Species ^a^	Formula	Experim.	Calculated	Error (ppm)
2:3	H_4_L_3_Zr_2_Na^+^	C_90_H_148_O_30_N_30_NaZr_2_	2331.88818	2331.89639	−3.52
2:3	H_4_L_3_Zr_2_Na^+^ −169	C_85_H_141_O_28_N_25_NaZr_2_	2162.93740	2162.83642	0.45
1:2	H_4_L_2_ZrNa^+^	C_60_H_100_O_20_N_20_NaZr	1533.63483	1533.63620	1.45
1:2	H_4_L_2_ZrNa^+^ −169	C_55_H_93_O_18_N_15_NaZr	1364.57874	1364.57623	1.84
1:1	HLZr^+^	C_30_H_49_O_10_N_10_Zr	799.26849	799.26747	1.28
1:1	LZrNa^+^	C_30_H_48_O_10_N_10_ZrNa	821.25037	821.24941	1.17
	H_4_LNa^+^	C_30_H_52_N_10_O_10_Na	735.37598	735.37677	0.790

^a^ Loss of 169 corresponds to a neutral C_5_H_7_N_5_O_2_ fragment, identified as Pm-NH_2_ (monoisotopic mass 169.0600).

**Table 3:** Comparison of models in [8,13] and refitting of data in [8] with the same hydrolysis model used in [13], either including (column 3) or excluding (column 4) non 1:1 complexes. Columns 5 and 6 contain synoptic views of selected log*K* values for Models 1 and 3, respectively.

Zr,H,DFO Species	1 logβ Ref [8]	2 logβ Ref [13]	3 Alternative logβ, Set 1	4 Alternative logβ, Set 2	5 log*K* ^a^ Column 1	6 log*K* ^a^ Column 3
101	36.02(9)	40.04(3)	40.95(9)	41.29(6)		
111	^b^	46.4(1)		49.01(7)		
121	49.06(6)		53.87(6)	51.8(1)	13.04 ^c^	12.92 ^c^
1-11	26.15(4)	29.15(9)	30.96(4)	30.6(1)	−9.87 ^c^	−9.99 ^c^
212	86.3(1)		95.9(1)		14.3 ^c^	14.0 ^c^
222	92.9(2)		102.55(2)		20.9 ^c^	20.6 ^c^
232	99.1(1)		108.7(1)		27.1 ^c^	26.8 ^c^
253	134.1(1)		143.7(1)		62.1 ^c^	61.8 ^c^
263	138.0(1)		147.6(1)		66.0 ^c^	65.7 ^c^

^a^ Log*K* values are computed for the formation of the Zr_x_H_y_DFO_z_ species and feature the association of as many ZrDFO species as possible, i.e., xZrDFO + yH + (z − x)DFO = Zr_x_H_y_DFO_z_. The only exception is the 1-11 species, whose log*K* corresponds to the 101 + H_2_O = 1-11 + H^+^ equilibrium. ^b^ If 2:2 species are neglected, a logβ = 44.7(1) is found for these species, with no modification in the log*K* value for 101 [8]. ^c^ Signals for the log*K* values among the models in Columns 1 and 3 are equal within the experimental error ranges.

## Data Availability

Not applicable.

## Supplementary Materials

The following are available online, Figure S1: Distribution diagrams of the species formed by DFO*–*Pm and DFO as a function of pH, Figure S2: pH dependence of Uv-vis spectra of DFO*–*Pm, Figure S3: Uv-vis spectral variations upon addition of Cu^2+^ to a DFO*–*Pm solution, Figure S4: Distribution diagram of the complexes formed by DFO*–*Pm with Cu^2+^, Figure S5: Comparison between the pH dependence of the absorbances of solutions containing DFO*–*Pm and Cu^2+^ in 1:1 and 1:2 molar ratios, Figure S6: UV-vis absorption spectra of a DFO*–*Pm solution upon addition of increasing amounts of Zn^2+^, Figure S7: Variation with equivalents of added Zn^2+^ or Zr^4+^ of the absorption of DFO*–*Pm at 327 nm, Figure S8: Isotopic distribution introduced by the presence of 1 or more Zr^4+^ ions, Figure S9: MS–MS spectra showing how the 2:3 peak is broken down into 1:2, 1:1, and Zr^4+^-free L, Figure S10: Outlook of experiment (a) in ref. [13], Figure S11: Outlook of experiment (b) in ref. [13], Figure S12: Outlook of experiment (c) in ref. [13].

## Sample Availability

Samples of the compounds used in this work are available from the authors.
