# Supplementary material for: Metal Coordination Properties of a Chromophoric Desferrioxamine (DFO) Derivative: Insight on the Coordination Stoichiometry and Thermodynamic Stability of Zr4+ Complexes
Source: Molecules. 2021 Dec 29;27(1):184. doi: 10.3390/molecules27010184 (PMC8746621; doi:10.3390/molecules27010184)
Supplement: Supplementary file 1 [file molecules-27-00184-s001.zip › molecules-1515297-supplementary.pdf]

Supplementary Material

Metal coordination properties of a chromophoric Desferrioxamine (DFO) derivative. Insight on the coordination stoichiometry and thermodynamic stability of Zr^{4+} complexes.

By Matteo Savastano, Francesca Boscaro and Antonio Bianchi

Fig. S1. Distribution diagrams of the species formed by DFO-Pm and DFO as a function of pH. …………… S2

Fig. S2. pH dependence of Uv-vis spectra of DFO-Pm. …………………………………………………………………………… S3

Fig. S3. Uv-vis spectral variations upon addition of Cu^{2+} to a DFO-Pm solution. ………………………………… S4

Fig. S4. Distribution diagram of the complexes formed by DFO-Pm with Cu^{2+}. ……………………………………. S5

Fig. S5. Comparison between the pH dependence of the absorbances of solutions containing DFO-Pm and Cu^{2+} in 1:1 and 1:2 molar ratios. ……………………………………………………………………………………………… S6

Fig. S6. UV-vis absorption spectra of a DFO-Pm solution upon addition of increasing amounts of Zn^{2+}.... S7

Fig. S7. Variation with equivalents of added Zn^{2+} or Zr^{4+} of the absorptions at 327 nm of DFO-Pm. …….. S8

Fig. S8. Isotopic distribution introduced by the presence of 1 or more Zr^{4+} ions. ……………………………… S9

Fig. S9. MS-MS spectra showing how the 2:3 peak is broken down into 1:2, 1:1 and Zr^{4+}-free L. ………… S10

Fig. S10. Outlook of experiment a) in ref. 13. ……………………………………………………………………………… S11

Fig. S11. Outlook of experiment b) in ref. 13. ……………………………………………………………………………… S12

Fig. S12. Outlook of experiment c) in ref. 13. ……………………………………………………………………………… S13

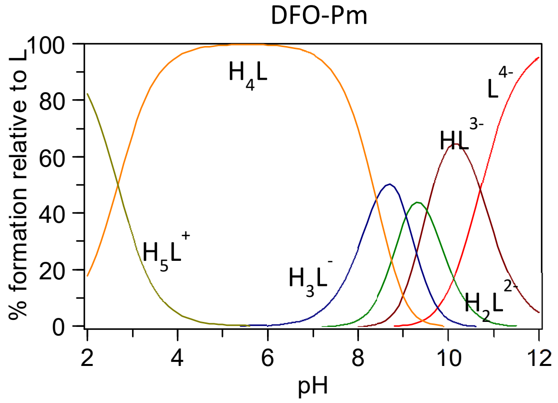

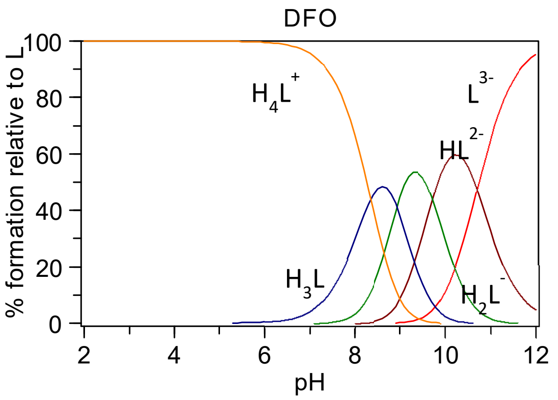

Figure S1. Distribution diagrams of the species formed by DFO-Pm (H_4L) and DFO (H_3L) as a function of pH. [DFP-Py] = [DFO] = 1×10^{-3} M.

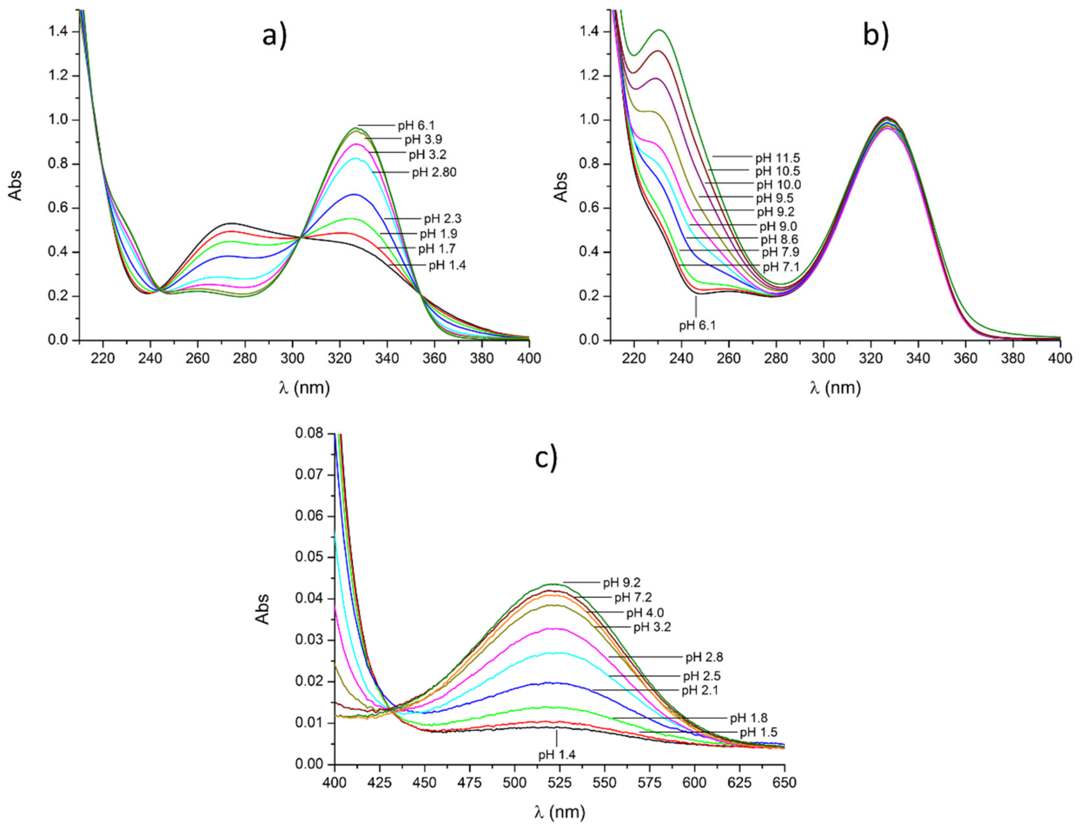

Figure S2. Uv-vis spectra of DFO-Pm in the pH ranges: a) 1.4-6.2, b) 6.1-11.5. c) Spectra in the Vis region, pH 1.5-9.2. [DFO-Pm] = 4.25×10^{-5} M.

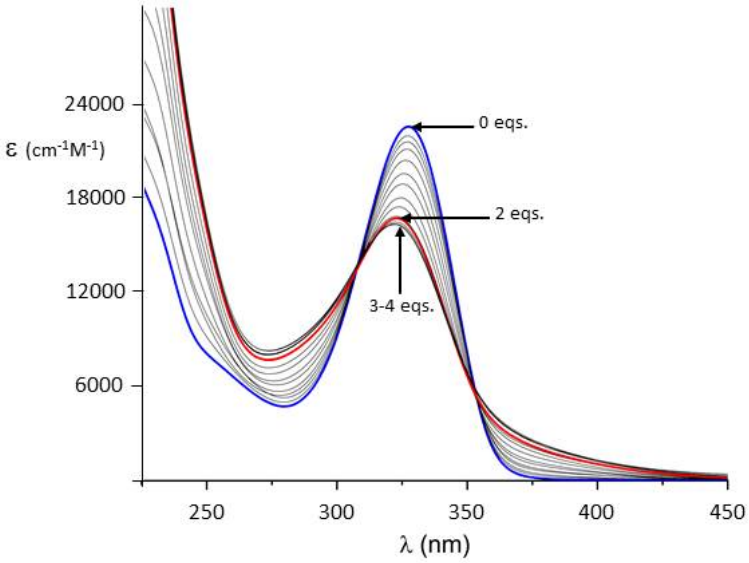

Figure S3. Uv-vis spectral variations upon addition of Cu^{2+} to a DFO-Pm solution (4.25×10^{-5}M, pH 6).

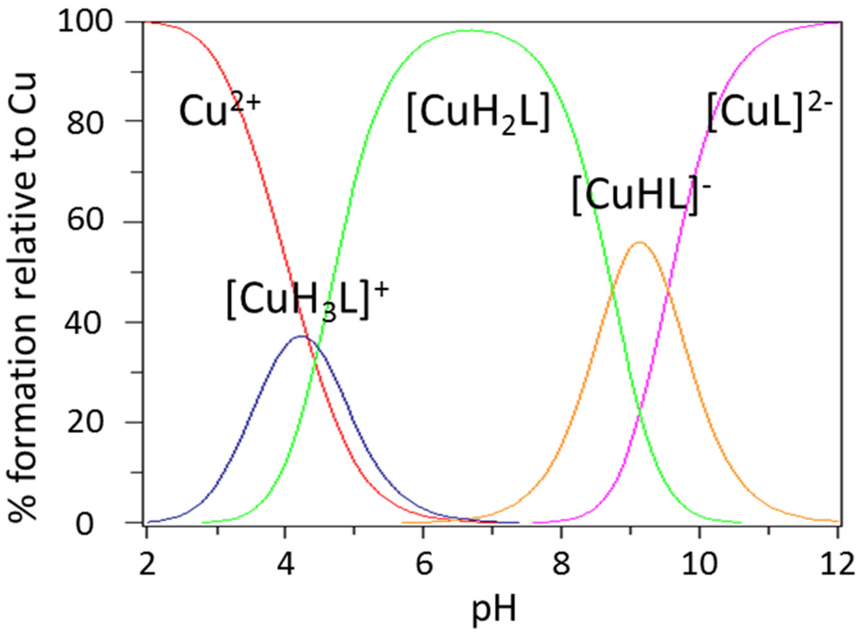

Figure S4. Distribution diagram of the complexes formed by DFO-Pm with Cu^{2+}. [DFO-Pm] = [Cu^{2+}] = 4.25×10^{-5} M.

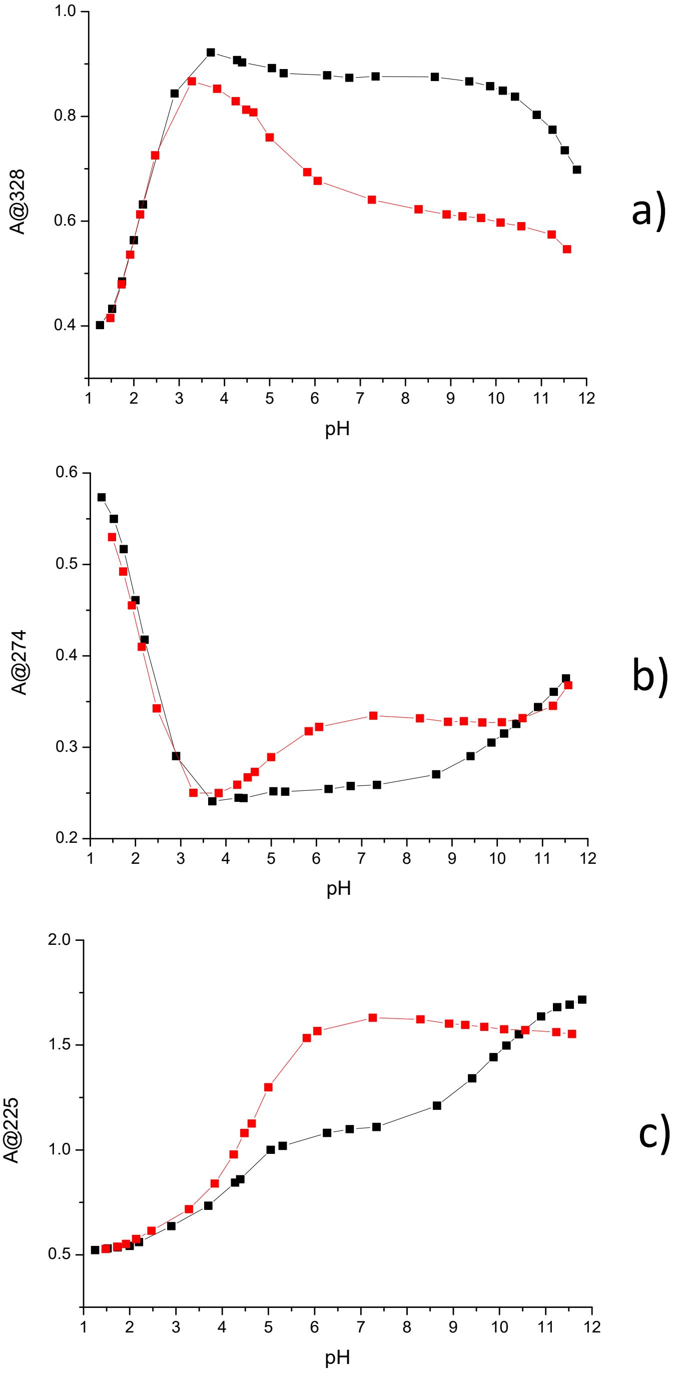

Figure S5. Comparison between the pH dependence of the absorbances at 328 nm (a), 274 nm (b) and 225 nm (c) of solutions containing DFO-Pm 4.25×10^{-5} M and Cu^{2+} 4.25×10^{-5} M (black squares) or 8.5×10^{-5} M (red squares).

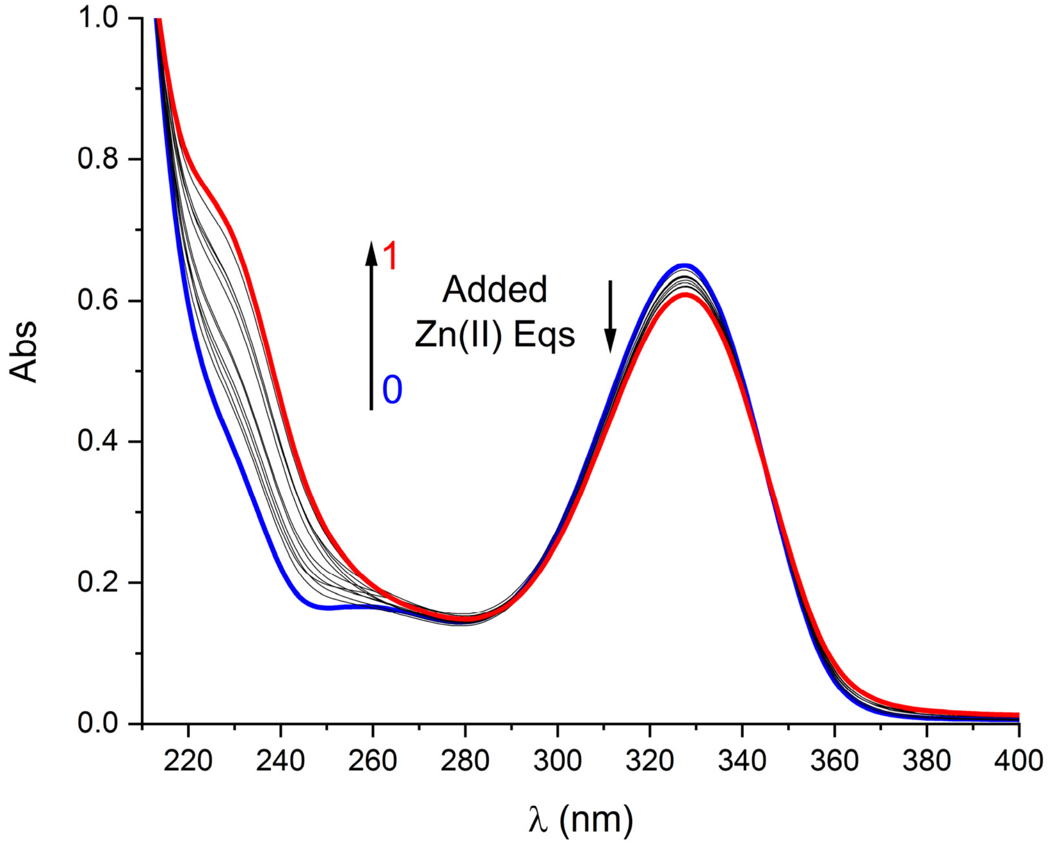

Figure S6. UV-vis absorption spectra of a DFO-Pm solution (3×10^{-5} M, pH 8) upon addition of increasing amounts of Zn^{2+}.

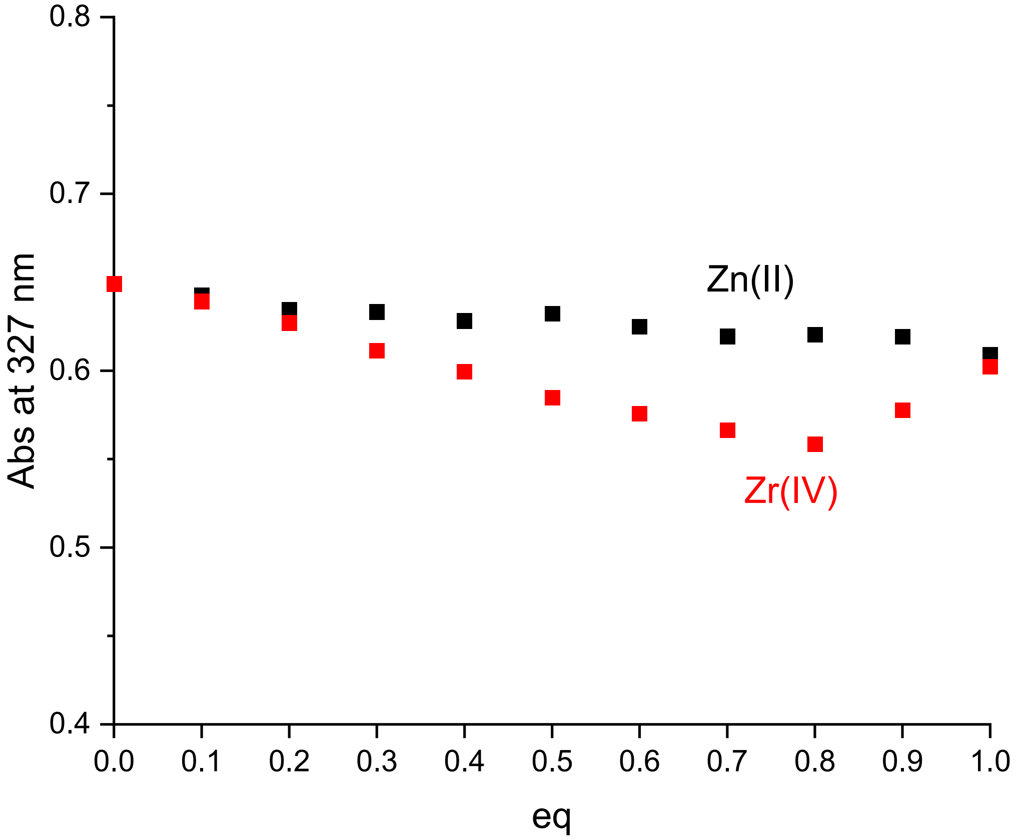

Figure S7. Variation with equivalents of added Zn^{2+} (pH 8.0) or Zr^{4+} (pH 6.0) of the absorptions at 327 nm. [DFO-Pm] = 3×10^{-5} M.

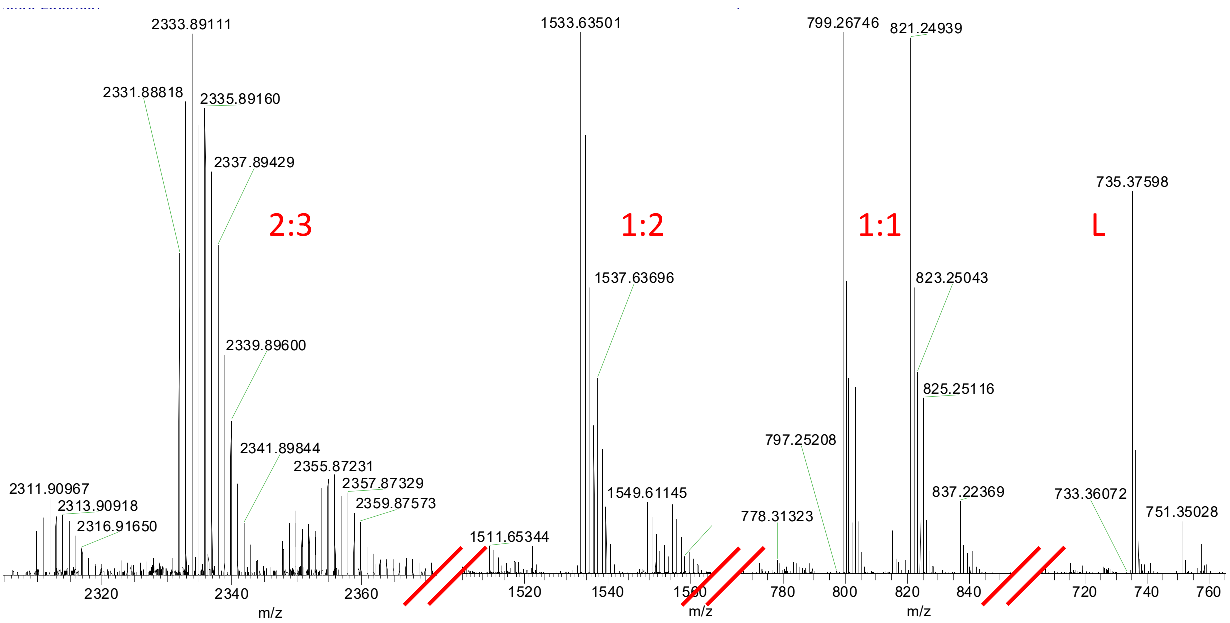

Figure S8. An example of the characteristic isotopic distribution introduced by the presence of 1 or more Zr(IV) ions, aiding peaks assignment.

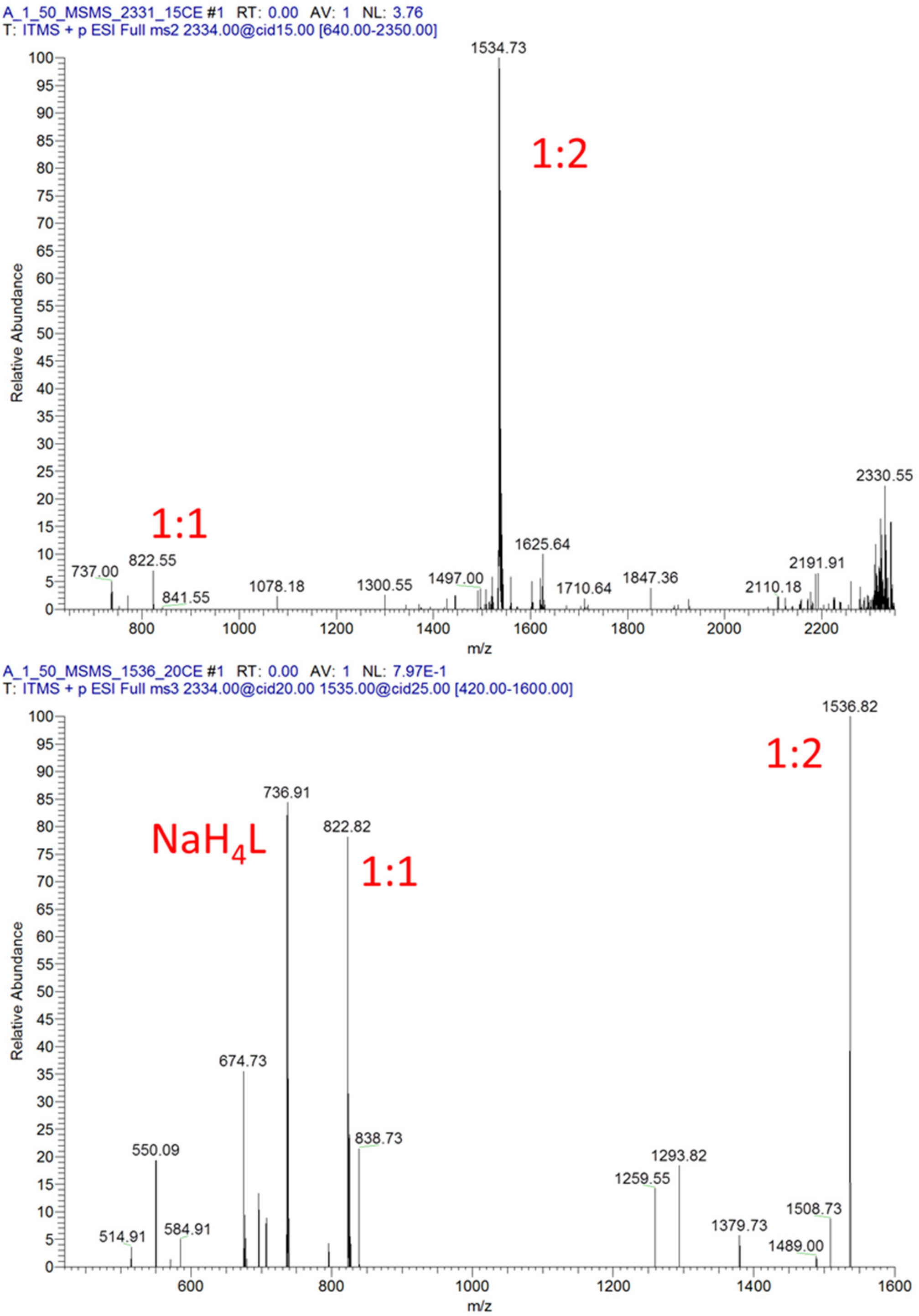

Figure S9. Top: MS-MS spectra showing how the 2:3 peak (m/z 2331) is broken down into 1:2, 1:1 and L not coordinated to Zr(IV). Bottom: 1:2 peak (m/z 1536) can be broken down into 1:1 and L not coordinated to Zr(IV) as well.

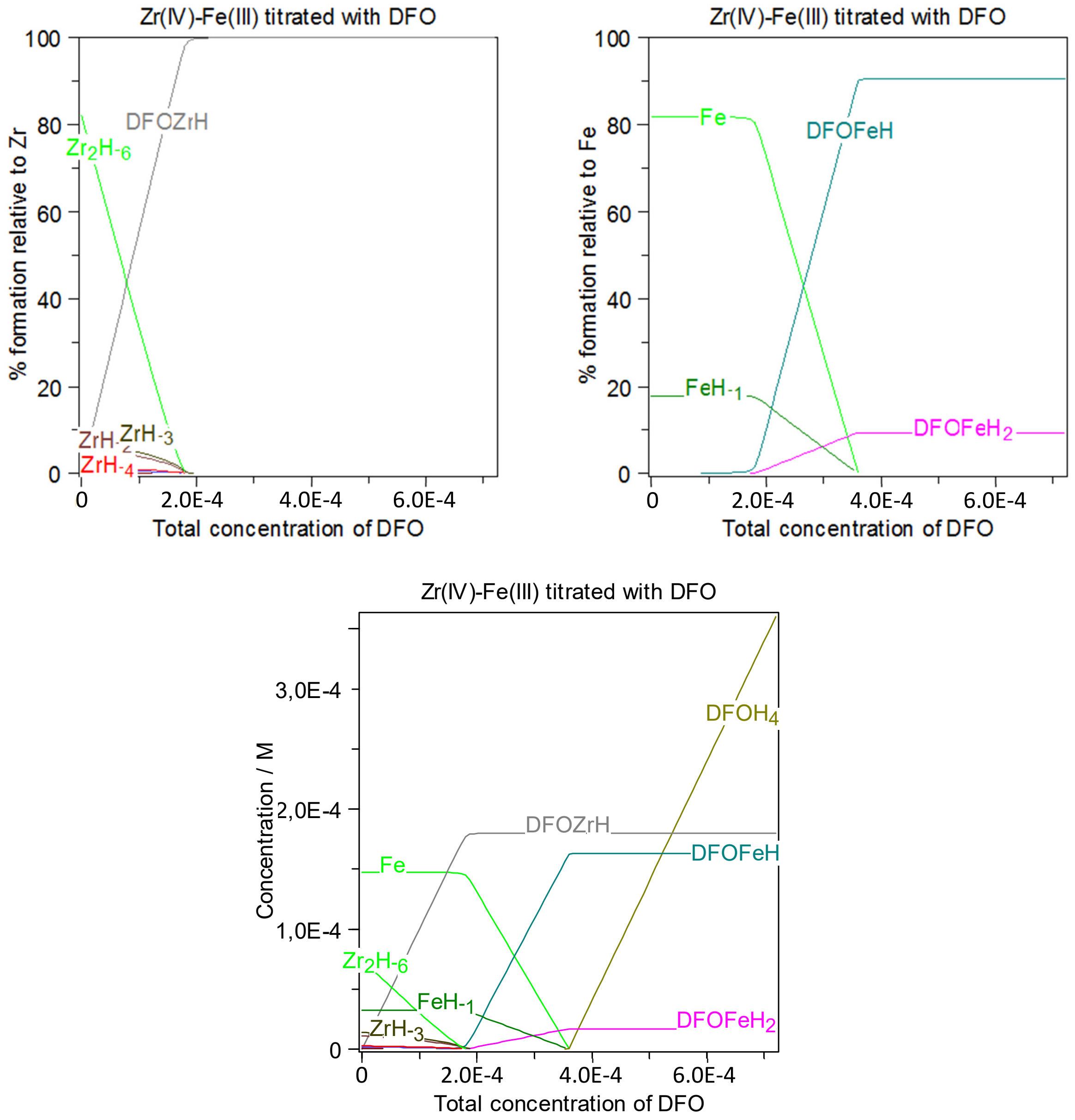

Figure S10. Outlook of experiment a): 1:1 Fe^{3+}: Zr^{4+} solution titrated with increasing equivalents of DFO (0 to 4 eqs). Concentrations and full thermodynamic set of constants from ref 13. Top left: the first equivalent of DFO selectively and exhaustively binds Zr^{4+} (no spectral changes involved). Top right: the second equivalent of DFO exhaustively binds Fe^{3+} (all spectral change must happen here). Bottom: global outlook of the experiment showing no Fe^{3+}/Zr^{4+} competition for DFO and end of the experiments at 2 added equivalents of DFO (last two equivalents of ligand remain free and cause no spectral change). Progressive spectral changes from 0 to 4 equivalents were instead reported (Figure 3 of ref. 13).

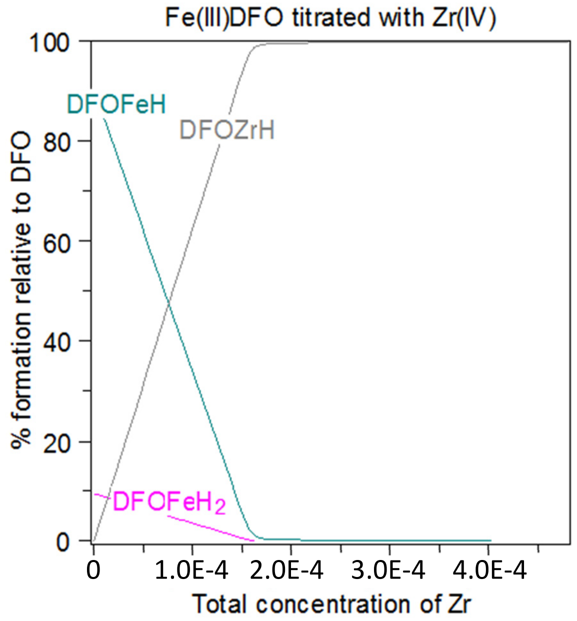

Figure S11. Outlook of experiment b): 1:1 Fe^{3+}:DFO solution titrated with Zr^{4+} (0 to 3 eqs). Concentrations and full thermodynamic set of constants from ref 13. 1 equivalent of Zr^{4+} should displace Fe^{3+} entirely causing discoloration of the solution. Spectral changes were instead observed up to 3 eqs of added Zr^{4+} (ref. 13). No Fe^{3+}/Zr^{4+} competition is present in any case: Zr^{4+} quantitatively displaces the Fe(III) complex.

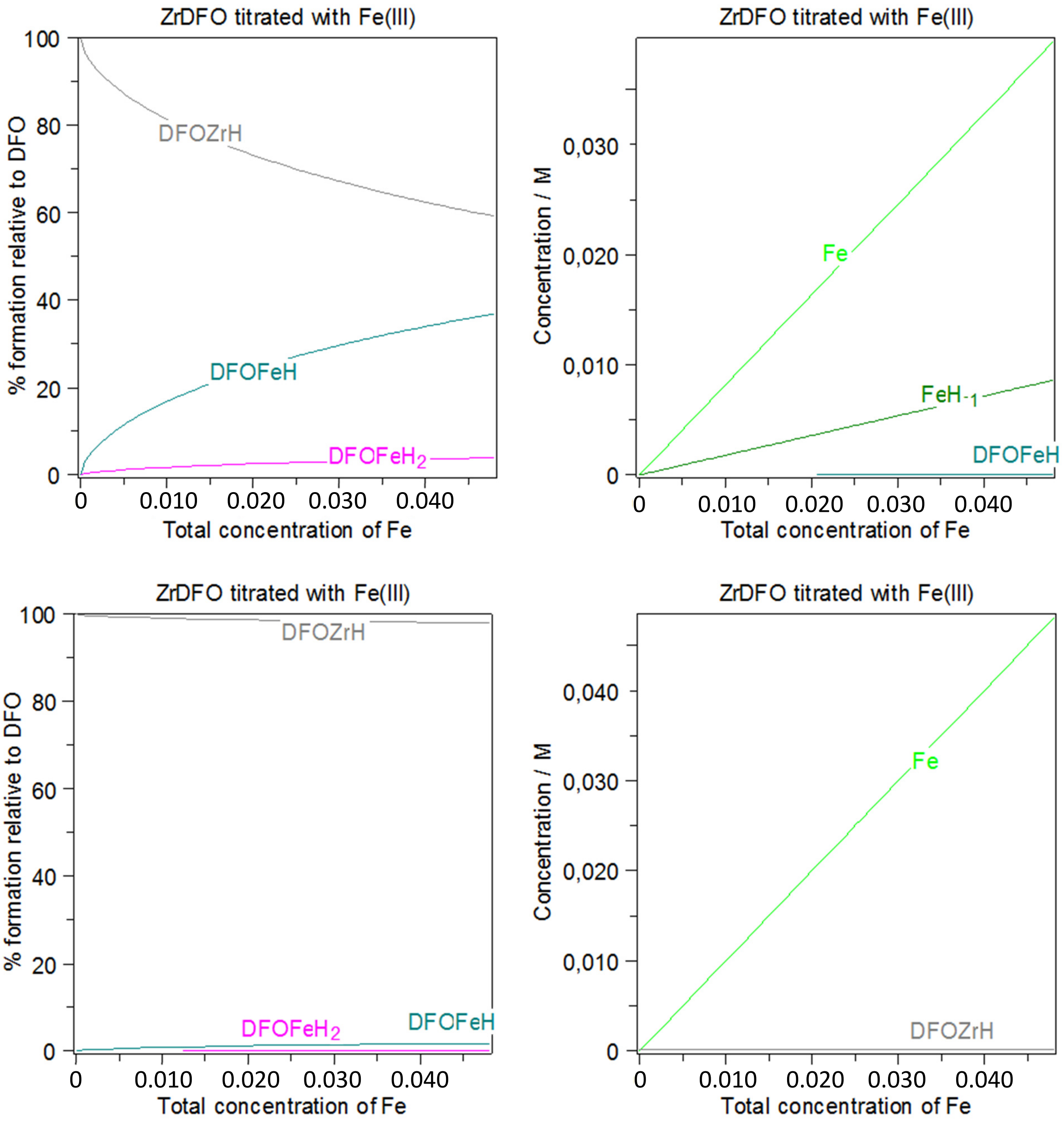

Figure S12. Outlook of experiment c): 1:1 Zr^{4+}:DFO solution titrated with Fe^{3+} (0 to 300 eqs). Concentrations and full thermodynamic set of constants from ref 13. Top: the real experiment displaying competition (the hydrolysis of Fe^{3+} and Zr^{4+} has been included in calculations). Bottom: same experiment ignoring hydrolytic equilibria for both metals: no dissociation of ZrDFO complex should occur, because Fe^{3+} does not compete with Zr^{4+} for DFO.
